# Microbial Aromatic Amino Acid Metabolism in Cardiometabolic Disease

**DOI:** 10.3390/nu18213563

**Published:** 2026-10-28

**Authors:** Jiyeon Kim, Ina Nemet

**Affiliations:** 1 Department of Heart, Blood, and Kidney Research, Cleveland Clinic Research, Cleveland Clinic, Cleveland, OH 44106, USA; kimj36@ccf.org; 2 Center for Microbiome & Human Health, Cleveland Clinic Research, Cleveland Clinic, Cleveland, OH 44106, USA; 3 Department of Molecular Medicine, Cleveland Clinic Lerner College of Medicine of Case Western Reserve University School of Medicine, Cleveland, OH 44106, USA

**Keywords:** gut microbiota, microbial metabolites, metabolomics, cardiovascular disease, metabolic disease, precision medicine, host-microbe interaction

## Abstract

The gut microbiota generates a diverse array of metabolites that influence host physiology. Among these, products of microbial metabolism of the aromatic amino acids tryptophan, phenylalanine, tyrosine, and histidine have emerged as important components of host-microbiome interactions relevant to cardiometabolic disease. This Review summarizes the microbial pathways, enzymes, and metabolic networks involved in aromatic amino acid metabolism and examines how dietary substrate availability, microbial community composition, microbial enzymatic capacity, host metabolism, and environmental factors influence metabolite production and biological activity. Microbiota-derived metabolites and host-microbial co-metabolites are associated with inflammation, vascular function, thrombosis, metabolic homeostasis, and host signaling pathways. Their biological effects are context-dependent and influenced by microbial pathway activity, host metabolic processing, tissue compartment, disease stage, and substrate availability. We further distinguish evidence derived from human observational studies, experimental metabolite administration, and demonstrated molecular mechanisms. Finally, we discuss strategies to modulate aromatic amino acid metabolism, including dietary interventions, functional foods, probiotics, fecal microbiota transplantation, microbial enzyme inhibitors, and metabolite sequestration approaches. Current evidence supports a framework linking dietary substrates, microbial enzyme activity, host responses, and cardiometabolic phenotypes, while highlighting important opportunities and challenges for developing precision microbiome-based therapeutics.

## 1. Introduction

The human gut microbiota is a complex, metabolically active ecosystem that influences host physiology through the production and transformation of numerous small molecules. The substrates and precursor pools supporting gut microbial metabolism arise from three principal sources: dietary components that escape digestion and absorption; host-derived materials, including mucins, shed epithelial cells, bile acids, and digestive secretions; and microbial biomass released through cell turnover or lysis, together with metabolites exchanged through microbial cross-feeding [1]. Metabolite production therefore reflects not only microbial community composition but also substrate availability, community functional potential, ecological conditions, and host physiology.

Short-chain fatty acids (SCFAs) generated by microbial fermentation of dietary fibers are among the most extensively characterized products of the gut microbiota [2,3]. Proteins that escape digestion and absorption in the upper gastrointestinal tract also reach the colon and support microbial metabolism. Amino acid fermentation generates structurally diverse compounds that may act locally, enter the portal or systemic circulation, or undergo further conversion by host enzymes. The resulting metabolite profile depends on the substrates reaching the colon, and on the organisms, enzymes, and physicochemical conditions present in the intestinal environment [4,5].

Within this three-source framework, the colonic protein pool includes undigested dietary protein and endogenous materials, such as mucins, shed epithelial cells, and pancreatic secretions, as well as microbial protein derived from cell turnover or lysis. Host digestive proteases and microbial extracellular proteases hydrolyze proteins and peptides into free amino acids [6], which are metabolized through reductive and oxidative pathways to generate ammonia, hydrogen sulfide, biogenic amines, aromatic compounds, and other small molecules.

Recent advances in isotope tracing and metaproteomics have begun to elucidate how microbial taxa access dietary and host-derived protein substrates in vivo. In the mouse model examined, most genera within Bacillota (formerly Firmicutes) preferentially utilized dietary protein, whereas host-derived mucins contributed substantially to microbial biomass at the community level [7]. Among protein-derived products, metabolites of the aromatic amino acids tryptophan (Trp), phenylalanine (Phe), and tyrosine (Tyr), together with the structurally related heteroaromatic amino acid histidine (His), are important mediators of host-microbial crosstalk. Metabolites derived from these amino acids arise through specialized pathways whose distribution and activity vary among microbial taxa and strains. Experimental studies have begun to identify the enzymes responsible for these transformations and to distinguish genetic potential from demonstrated activity.

Some of these amino acid-derived metabolites are generated directly by microorganisms, whereas others undergo host modification after absorption. The circulating concentrations of direct microbial products and host-microbial co-metabolites reflect microbial production, host metabolism, tissue distribution, and clearance. Metabolites within both groups can engage host receptors, including the aryl hydrocarbon receptor (AhR) and specific G protein-coupled receptors as well as nuclear receptors, thereby influencing immune responses, intestinal barrier function, vascular biology, and metabolism.

Despite growing recognition of their importance, key determinants of metabolite production in vivo remain incompletely defined. These include nutrient inputs, microbial interactions, strain-level variation in enzymatic capacity, host metabolic transformation, tissue distribution and clearance, and disease stage. Clarifying these factors is essential for interpreting metabolite measurements, establishing causality, and developing microbiome-based interventions.

In this Review, we examine microbial metabolism of Trp, Phe, Tyr, and His through a pathway-centered framework. We describe the enzymes and representative taxa involved, the ecological and host factors that govern pathway activity and metabolite disposition, and the links between these pathways and cardiometabolic phenotypes. We evaluate evidence from human observational studies, metabolite-administration experiments, microbial genetics, biochemical assays, cultured isolates, and animal models to distinguish associations from experimentally supported mechanisms. This approach highlights mechanistic gaps and opportunities for targeted modulation of microbial aromatic and heteroaromatic amino acid metabolism.

## 2. Microbial and Host Trp Co-Metabolism and Cardiometabolic Diseases

**Figure 1:**
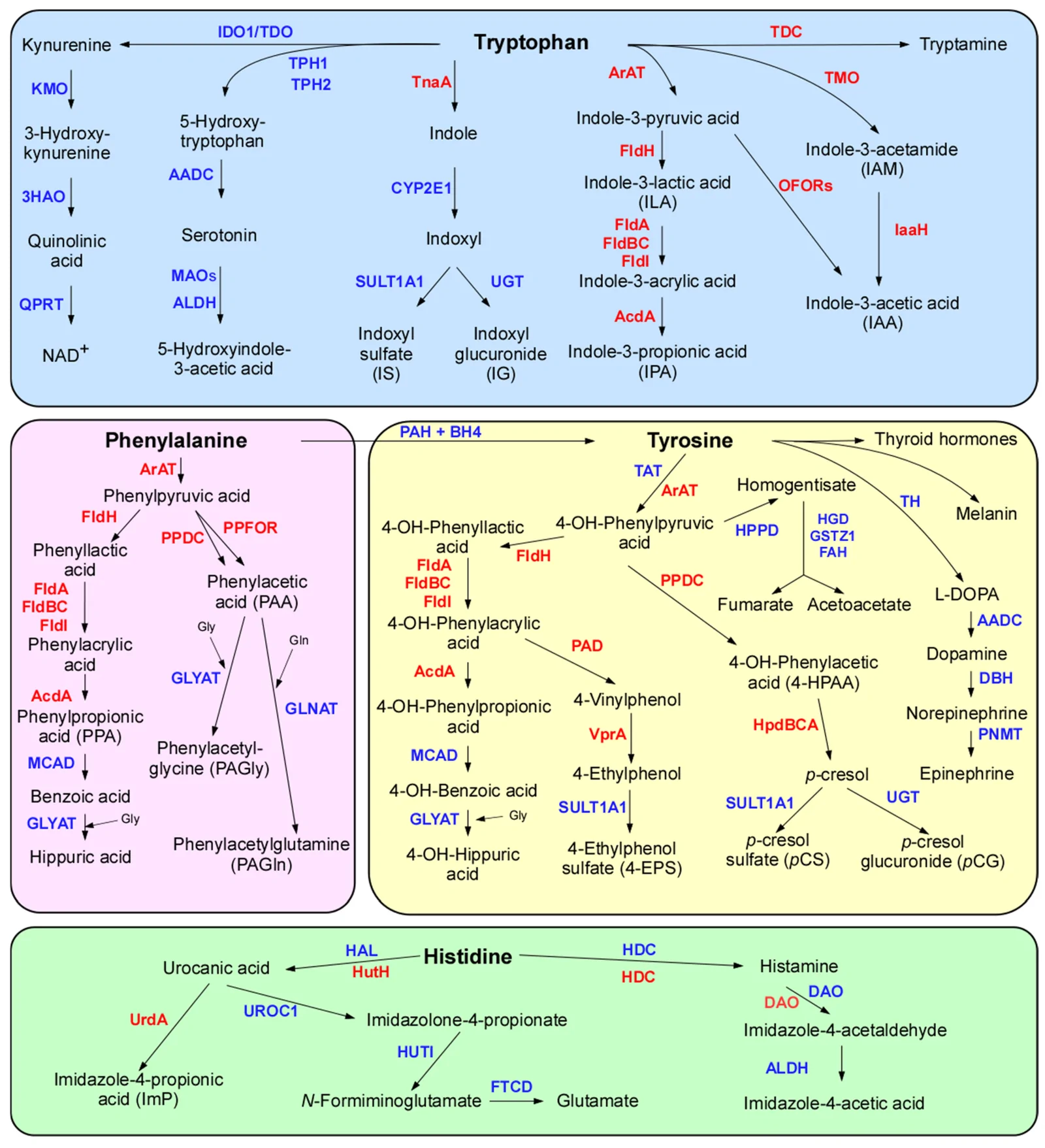
Host and microbial metabolism of aromatic amino acids. Schematic representation of major host and microbial pathways involved in tryptophan, phenylalanine, tyrosine, and histidine metabolism. Host-associated enzymes are shown in blue and microbial enzymes in red. Microbial products are generated through microbial pathways (red enzymes); following absorption, selected microbial products undergo further host metabolism (blue enzymes) to generate host-microbial co-metabolites. Supporting references and additional details regarding the pathways and experimental evidence are provided in the corresponding sections of the main text; abbreviations used in the figure are defined in the manuscript abbreviation list.

In the host, Trp is an essential aromatic amino acid that serves not only as a substrate for protein synthesis but also as a precursor for several bioactive metabolites, including serotonin and kynurenine-pathway intermediates, the latter of which ultimately contribute to nicotinamide adenine dinucleotide (NAD^+^) biosynthesis (Figure 1). The majority of host Trp catabolism occurs through the kynurenine pathway, initiated by indoleamine 2,3-dioxygenase 1 (IDO1), which is expressed in multiple extrahepatic tissues, including intestinal epithelial and immune cells, or by tryptophan 2,3-dioxygenase (TDO), which is expressed predominantly in the liver. Both enzymes catalyze the oxidation of Trp to *N*-formylkynurenine, which is subsequently converted through intermediates including 3-hydroxykynurenine to quinolinic acid, a precursor of NAD^+^ [8,9]. Peripheral serotonin (5-hydroxytryptamine; 5-HT) is synthesized predominantly by enterochromaffin cells in the gastrointestinal tract. Trp is first hydroxylated to 5-hydroxytryptophan (5-HTP) by tryptophan hydroxylase 1 (TPH1) in peripheral tissues or TPH2 in neurons, followed by decarboxylation of 5-HTP by aromatic L-amino acid decarboxylase (AADC). Serotonin is metabolized primarily by monoamine oxidase, followed by aldehyde dehydrogenase, to generate 5-hydroxyindole-3-acetic acid (5-HIAA) [10,11].

Although the kynurenine and serotonin pathways are predominantly host pathways, the intestinal microbiota can modulate their activity. Experimental studies indicate that microbial metabolites, including SCFAs and secondary bile acids such as deoxycholic acid, can regulate intestinal TPH1 expression and thereby influence host serotonin biosynthesis [8,9,10]. The microbiota can also modulate host IDO1 expression and activity [11,12]. These observations should be distinguished from direct microbial serotonin production. Earlier studies provided evidence that bacteria can produce or metabolize serotonin and related amines [13,14,15]. More recently, a consortium containing *Limosilactobacillus mucosae* and *Ligilactobacillus ruminis* was shown to produce bioactive serotonin in vitro by decarboxylating 5-HTP. Colonization of germ-free *Tph1*^−/−^ mice with these bacteria increased fecal serotonin, promoted colonic innervation, and altered intestinal transit, providing in vivo evidence in mice for a direct microbial contribution to intestinal serotonin pools. In the human component of the study, a lower relative fecal abundance of *L. mucosae* was observed in individuals with irritable bowel syndrome [16]; however, this association does not establish that microbial serotonin production makes a quantitatively important contribution to serotonin pools in humans. Thus, microbial regulation of host serotonergic metabolism and direct microbial production of serotonin should be regarded as related but mechanistically distinct processes.

Beyond modulation of host pathways, gut microorganisms directly convert Trp into a structurally diverse group of indole derivatives and other small molecules (Figure 1, Table 1). Tryptamine production has been demonstrated in vitro in several human intestinal bacterial taxa, including *Clostridium sporogenes*, *Mediterraneibacter gnavus* (formerly *Ruminococcus gnavus*), *Blautia hansenii*, *Enterocloster asparagiformis*, and *Faecalimonas nexilis* (formerly *Clostridium nexile*), although production capacity varies among organisms and experimental conditions. Biochemical studies have identified and characterized bacterial tryptophan decarboxylases (TDCs) that catalyze the conversion of Trp to tryptamine, while metagenomic analyses demonstrate the presence of TDC-encoding genes in human gut microbiomes [17,18,19,20]. Causality has also been demonstrated in vivo in gnotobiotic mice using genetically matched bacterial strains. Germ-free mice were colonized with an engineered *Bacteroides thetaiotaomicron* strain expressing a bacterial TDC or with a corresponding control strain lacking tryptamine-producing capacity. Colonization with the TDC-expressing strain increased luminal tryptamine, demonstrating that bacterial TDC activity can influence host intestinal physiology by increasing 5-HT4 receptor-dependent epithelial secretion and accelerating gastrointestinal transit [19]. However, the relative contributions of naturally occurring TDC-producing taxa to intestinal or circulating tryptamine concentrations in humans remain incompletely defined.

A separate microbial pathway is catalyzed by the pyridoxal 5′-phosphate (PLP)-dependent enzyme tryptophanase (TnaA), encoded by *tnaA*, which converts Trp to indole, pyruvate, and ammonia [21]. After intestinal absorption, indole is transported through the portal circulation and undergoes sequential host metabolism. In hepatocytes, indole is oxidized to indoxyl, principally by cytochrome P450 2E1 (CYP2E1). Indoxyl is subsequently conjugated by hepatic phase II enzymes: sulfotransferase 1A1 (SULT1A1) catalyzes sulfation to form indoxyl sulfate (IS), whereas UDP-glucuronosyltransferases (UGTs) catalyze glucuronidation to form indoxyl glucuronide (IG) [22,23,24] (Figure 1, Table 1). Thus, formation of IS and IG represents host-microbial co-metabolism, and circulating IS and IG concentrations may reflect bacterial indole production together with intestinal absorption, hepatic biotransformation, and renal clearance.

Microbial transamination of Trp generates indole-3-pyruvate, which can enter a reductive pathway leading to indole-3-propionic acid (IPA). This pathway was mechanistically characterized in *C. sporogenes* by Dodd et al. [25]. Indole-3-pyruvate is reduced to indole-3-lactic acid (ILA) by FldH, a phenyllactate/aryl-lactate dehydrogenase, followed by reactions involving FldA, an acyl-CoA transferase; FldBC, the aryl-lactoyl-CoA dehydratase complex; its activating enzyme FldI; and AcdA, an acyl-CoA dehydrogenase involved in the terminal reductive step (Figure 1, Table 1). Dodd et al. generated *fldH*, *fldC*, and *acdA* mutants and demonstrated in vitro that disruption of these genes impaired reductive Trp metabolism and IPA production. They further demonstrated the relevance of this pathway in vivo using the *fldC* mutant in gnotobiotic mice. Liu et al. [26] subsequently extended these findings by directly examining IPA production in the host: *fldC* and *acdA* mutants were defective in IPA production in vitro, and mice colonized with the *fldC* mutant had undetectable plasma IPA despite colonization levels comparable to the wild-type strain, providing direct genetic evidence that this bacterial pathway generates circulating IPA in vivo.

An alternative route of microbial Trp metabolism involves OFOR-mediated oxidative decarboxylation of indole-3-pyruvate to indoleacetyl-CoA, a putative intermediate in indole-3-acetic acid (IAA) formation. Screening of 206 human gut bacterial isolates identified two acetogens, *Intestinibacter bartlettii* and *Blautia hydrogenotrophica* as high-capacity IAA producers and implicated 2-oxoacid:ferredoxin oxidoreductases (OFORs) in this anaerobic pathway [27]. In vitro bacterial culture, genomic analyses, and heterologous enzyme assays demonstrated OFOR activity toward aromatic 2-oxoacids, including conversion of indole-3-pyruvate to indoleacetyl-CoA. In these gut acetogens, amino acid oxidation is linked to cellular redox metabolism, with CO_2_ released during oxidation reassimilated into acetate through reductive acetogenesis. Production of amino acid-derived metabolites was also demonstrated in vivo in gnotobiotic mice monocolonized with *I. bartlettii* [27]. Although the identification of high-capacity IAA producers among human gut isolates supports the potential relevance of this pathway in humans, its quantitative contribution to intestinal or circulating IAA remains undetermined.

IAA can also be generated through the indole-3-acetamide (IAM) pathway, a mechanistically distinct two-step route characterized primarily in plant-associated bacteria, such as *Agrobacterium tumefaciens* and *Pseudomonas savastanoi* [28]. In the first step, the flavin-dependent enzyme tryptophan 2-monooxygenase (TMO), encoded by *iaaM* and also referred to as *tms1*/*aux1*, catalyzes the oxidative decarboxylation of Trp to IAM. In the second step, indole-3-acetamide hydrolase, encoded by *iaaH* and also referred to as *tms2*/*aux2*, hydrolyzes IAM to IAA, releasing ammonia. Because TMO requires molecular oxygen, evidence obtained from these organisms should not be extrapolated directly to the predominantly anaerobic environment of the colonic lumen.

Nevertheless, an *iaaM*-dependent IAA production mechanism has been experimentally demonstrated in the gut-associated bacterium *Limosilactobacillus reuteri*. In vitro, wild-type *L. reuteri* produced IAA, whereas deletion of *iaaM* impaired IAA production. In mouse models, administration of wild-type *L. reuteri* promoted mucin sulfation and protection against experimental colitis, whereas the *iaaM*-deficient strain failed to reproduce these effects. Cell-based and mouse experiments further implicated IAA-dependent AhR signaling in regulation of the mucin-sulfation pathway [29]. These findings provide strain-specific mechanistic evidence linking bacterial *iaaM*-dependent IAA production to intestinal effects in experimental systems, but they do not establish that this pathway is widespread or quantitatively important in the human intestinal microbiome (Figure 1, Table 1).

In summary, microbial Trp metabolism generates a network of bioactive metabolites through biochemically distinct and taxon-dependent pathways. Reductive metabolism can generate ILA and IPA, whereas oxidative pathways can contribute to IAA and other indole derivatives. The strength of mechanistic evidence differs among these pathways: several reactions have been characterized in bacterial cultures and evaluated in animal models, whereas the contributions of individual organisms and enzymes to metabolite production in humans remain less clearly defined.

### 2.1. Indole, IS, and IG in Cardiometabolic Diseases

IS and IG are host-conjugated metabolites of microbiota-derived indole. Accordingly, they are more precisely described as host-microbial co-metabolites rather than exclusively microbial metabolites. They are commonly classified as “uremic toxins” because they accumulate with declining renal function and have been implicated in the pathogenesis of adverse systemic phenotypes in chronic kidney disease (CKD) and end-stage kidney disease (ESKD) [30]. However, the clinical and mechanistic evidence is substantially more extensive for IS than for IG. Patients with CKD have a markedly increased burden of cardiovascular disease (CVD) and mortality [31,32]. Clinical studies, meta-analyses, and reviews have associated elevated circulating IS concentrations with cardiovascular events and all-cause mortality in patients with impaired renal function [33,34,35].

In hemodialysis populations, IS has been extensively studied as a biomarker and potential mediator of cardiovascular risk. Higher circulating IS levels have been associated with cardiovascular events, mortality, and cardiovascular and mineral-metabolism abnormalities [36,37,38]. However, these associations are not entirely consistent across all cohorts; notably, in participants from the Hemodialysis (HEMO) study, a large U.S. multicenter trial, IS levels were not significantly associated with cardiovascular outcomes [39], highlighting heterogeneity across populations and study designs.

Beyond traditional cardiovascular endpoints, IS is increasingly associated with non-classical risk phenotypes in dialysis populations, including sarcopenia and increased hospitalization risk [40,41]. These relationships often persist after adjustment for conventional cardiovascular risk factors, but residual confounding remains possible, particularly because IS concentrations are closely related to renal clearance and the severity of kidney dysfunction. Therefore, these observations do not establish protein-bound uremic toxins as CKD-specific causal drivers of residual risk. The limited dialytic clearance of albumin-bound solutes such as IS likely contributes to their accumulation and may partly explain the stronger associations observed in ESKD compared with earlier stages of CKD.

Emerging evidence suggests that the clinical relevance of IS may extend beyond kidney disease. Recent studies have associated higher circulating IS concentrations with increased mortality risk in broader clinical populations not restricted to patients with advanced CKD, raising the possibility that IS may reflect pathophysiological processes related to systemic inflammation, microbial metabolism, or cardiovascular dysfunction [42,43]. However, these observational associations cannot determine whether IS contributes directly to adverse outcomes or instead acts as a marker of altered renal clearance, microbial metabolism, systemic disease, or combinations of these factors.

**Figure 2:**
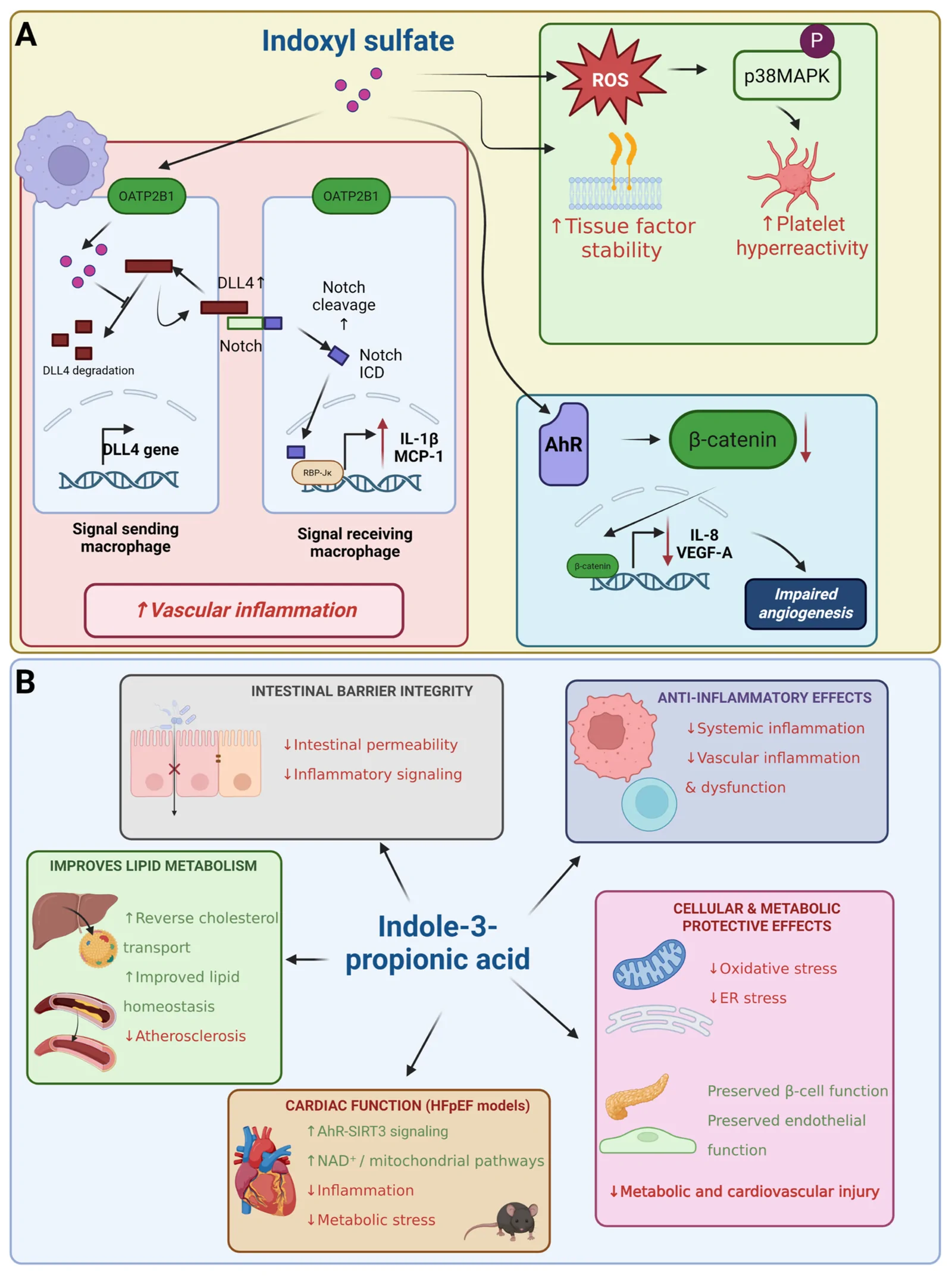
Microbial tryptophan metabolites in cardiometabolic disease. Experimentally supported effects of (**A**) the host-microbial co-metabolite indoxyl sulfate on thrombosis, vascular inflammation, and angiogenesis and (**B**) the microbial metabolite indole-3-propionic acid on intestinal barrier function, inflammation, lipid metabolism, cellular and metabolic protection, and cardiac function. Evidence is derived from in vitro studies and in vivo animal models. Supporting references and details of the experimental models are provided in the corresponding sections of the main text.

In contrast to the predominantly associative human evidence, experimental studies provide mechanistic evidence for direct thrombotic and vascular effects of IS (Figure 2A, Table 2). In cellular and animal models, IS enhances platelet activity through reactive oxygen species (ROS)-mediated p38 mitogen-activated protein kinase (p38 MAPK) signaling [44], alters tissue factor stability by inhibiting its ubiquitination [45], and enhances thrombus formation in animal models of vascular injury [42,44,46]. Mechanistically, IS exerts pleiotropic effects on the vasculature, including induction of oxidative stress, endothelial dysfunction, and activation of pro-inflammatory signaling pathways such as nuclear factor κB (NF-κB), alongside reduced nitric oxide bioavailability [47,48]. These experimental mechanisms may help explain the clinical associations observed in CKD but have not been established as mediators of cardiovascular events in humans.

In addition, IS functions as a ligand for the AhR, driving transcriptional programs that alter angiogenesis and vascular repair in experimental systems. Chronic IS-mediated AhR activation impaired endothelial-cell tube formation and proliferation and reduced sprouting in aortic-ring cultures; genetic or pharmacological inhibition of AhR rescued these effects [49,50]. However, the effects of IS on angiogenesis appear to be context-dependent. In a three-dimensional human umbilical vein endothelial cell (HUVEC)–pericyte co-culture model, IS enhanced angiogenic responses, including increased tubule formation, while silencing of cytochrome P450 family 1 subfamily B member 1 (CYP1B1) reduced both IS-induced ROS production and the angiogenic response, implicating CYP1B1-dependent ROS signaling in this effect [51]. These divergent findings may reflect differences in experimental models, cell types, and exposure conditions and indicate that the vascular effects of IS are not uniformly anti-angiogenic.

IS also promotes pro-inflammatory macrophage activation through interplay between organic anion-transporting polypeptide 2B1 (OATP2B1) and DLL4–Notch signaling. In human macrophages, exposure to clinically relevant concentrations of IS promoted pro-inflammatory activation, and mechanistic studies implicated OATP2B1-mediated IS uptake and identified Notch signaling as a downstream pathway; Notch activation was preceded by rapid induction of DLL4 protein. These findings were further validated in vivo in murine CKD and atherosclerosis models, in which macrophage-targeted OATP2B1/*Slco2b1* silencing and Dll4 blockade or silencing attenuated IS-induced pro-inflammatory macrophage activation and atherosclerotic lesion development [52]. Separately, experimental studies have linked IS to vascular remodeling and calcification, including vascular smooth muscle cell (vSMC) dysfunction and vascular and valvular calcification [53,54] (Figure 2A, Table 2).

In the context of obesity and metabolic syndrome, IS is positively associated with waist-to-hip ratio in individuals with stable coronary artery disease (CAD) [55]. Reviews of experimental evidence suggest that IS and other uremic toxins may impair insulin signaling in CKD, whereas clinical studies mainly demonstrate associations rather than causality [56,57,58]. Accordingly, the independent causal contribution of IS to obesity, insulin resistance, or metabolic syndrome in humans remains unresolved.

While IS is a circulating toxin linked to cardiometabolic and renal pathology, these predominantly detrimental systemic effects contrast with the biological activity of its precursor, indole, within the gut. Indole plays a role in maintaining intestinal barrier integrity and mucosal homeostasis. In vitro studies using intestinal epithelial cells have shown that indole can attenuate inflammatory barrier dysfunction, increase the expression of tight-junction proteins, and suppress NF-κB signaling [59]. Indole exposure was also associated with increased AhR and CYP1A1 expression, supporting involvement of AhR signaling in these epithelial responses [59]. These findings suggest that the biological consequences of microbial Trp metabolism depend strongly on metabolite identity and physiological compartment, with intestinal indole displaying barrier-supportive activities that contrast with the adverse systemic effects associated with its host-derived metabolite IS.

### 2.2. IPA, Clinical Associations and Experimental Mechanisms

Multiple prospective and cross-sectional human cohort studies associate higher circulating IPA concentrations with a more favorable metabolic phenotype and lower risk of type 2 diabetes (T2D). In the Finnish Diabetes Prevention Study, higher serum IPA was associated with a lower incidence of T2D and with preservation of insulin secretion, while IPA concentrations were also inversely associated with high-sensitivity C-reactive protein (hsCRP), a marker of low-grade inflammation [60]. These findings are supported by additional high-risk cohorts in which circulating IPA was associated with T2D risk and low-grade inflammation [61]. Recent multi-omics analyses further link variation in circulating IPA to metabolic phenotypes, including obesity and insulin resistance [62]. Collectively, these human studies support IPA primarily as a biomarker of metabolic health and T2D risk; whether IPA itself contributes causally to glucose homeostasis cannot be established from these observational data. Experimental studies provide evidence that IPA can influence several metabolic and inflammatory pathways, supporting investigation of IPA as a potential functional mediator.

Extending these observations to liver disease, lower circulating IPA has been associated with adverse hepatic phenotypes, including liver fibrosis. In individuals undergoing bariatric surgery with detailed liver histologic assessment, circulating IPA concentrations were lower in those with fibrosis, with the association being particularly evident among individuals without T2D [63], whereas higher circulating IPA has been associated with greater gut microbial diversity [64]. Together, these observational findings link circulating IPA with metabolic and hepatic health, although they do not establish a causal relationship.

Clinical and metabolomic studies also associate circulating IPA with cardiovascular outcomes and atherosclerotic cardiovascular disease. In a large cohort of participants undergoing elective diagnostic cardiac evaluation, IPA was inversely associated with all-cause mortality but not with major adverse cardiac events (MACE) [43]. Among patients with established CAD, higher circulating IPA levels were prospectively associated with lower risks of both all-cause and cardiovascular mortality [65]. Similarly, metabolomic analyses identified lower circulating IPA in patients with CAD and an association between IPA and prevalent atherosclerotic cardiovascular disease (ASCVD) and disease severity [66]. A similar distinction is important for heart failure with preserved ejection fraction (HFpEF). Circulating IPA levels were reduced in two independent human HFpEF cohorts [67].

Across experimental and human studies, several mechanisms may underlie the associations between IPA and cardiometabolic health. IPA has been implicated in maintaining intestinal barrier integrity, with potential consequences for systemic inflammatory signaling [68]. Circulating IPA levels have also been associated with dietary and microbiome-related factors, supporting its role as an interface between diet, microbial metabolism, and host physiology [64,69]. Experimental evidence further supports anti-inflammatory and antioxidant actions of IPA and effects on cellular stress and mitochondrial homeostasis [61,70,71,72,73]. In atherosclerosis models, IPA promotes macrophage cholesterol efflux and reverse cholesterol transport through the microRNA-142-5p (miR-142-5p)/ATP-binding cassette transporter A1 (ABCA1) pathway [74]. In HFpEF models, IPA influences the AhR–sirtuin 3 (SIRT3) axis and NAD^+^ metabolism, providing a distinct potential mechanism for its effects on cardiac function [67] (Figure 2B, Table 2). However, these mechanistic effects should not be assumed to account directly for associations observed in human cohorts unless mediation has been demonstrated.

Together, these findings position IPA as a microbiota-derived metabolite associated with favorable cardiometabolic phenotypes in human cohorts, while cell and animal studies provide evidence for direct effects on glucose metabolism, hepatic fibrogenic pathways, atherosclerosis, and cardiac function. However, IPA supplementation has not consistently reproduced these protective effects across experimental models [75], indicating that the biological activity of IPA may depend on disease context, dose, timing, tissue exposure, dietary substrate availability, and the broader microbial ecosystem. Thus, circulating IPA may reflect both the activity of the metabolite itself and the functional state of the diet-microbiota-host axis, rather than acting as an isolated and universally protective mediator.

### 2.3. Other Microbial Metabolites Derived from Trp in Cardiometabolic Diseases

In the EPIC-Norfolk prospective population study of 11,972 individuals followed for a median of 22.1 years, higher circulating Trp levels were associated with lower all-cause mortality and fatal CVD, whereas higher kynurenine levels and an elevated kynurenine/Trp ratio were associated with increased risk of incident CVD. Serotonin was not associated with overall CVD but showed associations with myocardial infarction and stroke. Adjustment for C-reactive protein (CRP) did not appreciably alter these associations, suggesting that they were not explained by this marker of systemic inflammation [76]. In a large study combining participants from the U.S. and Europe, higher Trp levels were also linked to lower risk of MACE and death. In the same study, higher serotonin levels initially appeared to be associated with lower MACE risk; however, this association was no longer statistically significant after adjustment for established cardiovascular risk factors [43].

Among other Trp-derived metabolites, IAA illustrates a particularly context-dependent pattern. In CKD, circulating IAA accumulates, and higher concentrations have been associated with cardiovascular events and mortality. In cultured endothelial cells, IAA induces oxidative stress and activates pro-inflammatory AhR/p38 MAPK/NF-κB signaling, providing a potential mechanism linking IAA accumulation to vascular injury [77,78,79].

In contrast, impaired microbial production of AhR-active tryptophan metabolites, including IAA and indole-3-aldehyde (IAld), was observed in individuals with obesity and T2D [80]. Experimental studies in metabolic disease models have reported beneficial effects of IAA. In a Western diet-induced MASLD mouse model, IAA administration attenuated hepatic steatosis and inflammation, accompanied by suppression of NF-κB signaling, reduced endotoxin levels, and reduced macrophage activation [81,82]. Thus, the apparently opposing actions of IAA likely depend on physiological and disease context, including its accumulation as a uremic solute in CKD versus its activity as a microbiota-derived metabolite in experimental metabolic disease. Related microbial indole aldehydes have also shown protective effects in experimental models of obesity and inflammatory liver disease, with AhR signaling implicated in some settings [83,84,85].

In summary, Trp-derived metabolites exemplify the context-dependent nature of aromatic amino acid metabolism. Indole-derived uremic solutes such as IS and IAA in the setting of impaired renal clearance are associated with adverse vascular and renal phenotypes, whereas IPA and selected AhR-active indole derivatives show protective effects in several metabolic and inflammatory experimental settings. These contrasting effects emphasize that biological activity cannot be inferred from the indole structure alone but depends on the specific metabolite, concentration, tissue compartment, disease state, and experimental context.

## 3. Microbial and Host Phe Co-Metabolism and Cardiometabolic Diseases

In humans, Phe metabolism occurs predominantly in the liver, where phenylalanine hydroxylase (PAH) irreversibly hydroxylates Phe to Tyr. This reaction requires molecular oxygen and tetrahydrobiopterin (BH_4_), which is oxidized during the reaction and subsequently regenerated by dihydropteridine reductase (DHPR) [86,87] (Figure 1).

Gut microbial metabolism of Phe is initiated primarily by transamination to phenylpyruvate, which serves as a branch-point intermediate for the production of phenylacetic acid (PAA) and phenylpropionic acid (PPA) (Figure 1, Table 1). Two distinct microbial pathways for converting phenylpyruvate to PAA have been characterized. In the first, phenylpyruvate:ferredoxin oxidoreductase (PPFOR) catalyzes the oxidative decarboxylation of phenylpyruvate. In the second, phenylpyruvate decarboxylase (PPDC) catalyzes non-oxidative decarboxylation to phenylacetaldehyde, which is subsequently oxidized to PAA. Genetic studies in bacterial cultures identified PPFOR as a major contributor to PAA production in *B. thetaiotaomicron* and PPDC as an important contributor in *Proteus mirabilis*. In vivo evidence was obtained in gnotobiotic mice colonized with the corresponding bacterial strains [88].

Phe can also undergo reductive microbial metabolism to PPA through a pathway shared with other aromatic amino acids. Phenylpyruvate is first reduced to phenyllactate by FldH. Subsequent steps involve FldA, FldBC, FldI, and AcdA [25]. The pathway has been characterized experimentally in *C. sporogenes* using bacterial culture and genetic approaches, with additional support from colonized mouse models [25]. The extent to which individual organisms or enzymes contribute to PPA generation in humans remains incompletely defined.

Following intestinal production and absorption, PAA and PPA undergo further host metabolism. PAA is conjugated predominantly with glutamine in humans to generate phenylacetylglutamine (PAGln), whereas glycine conjugation to phenylacetylglycine (PAGly) predominates in rodents [89]. Thus, PAGln and PAGly are host-microbial co-metabolites formed through sequential microbial PAA production and host conjugation. Experimental studies using bacterial genetics and gnotobiotic mice directly support this meta-organismal pathway [88,90].

PPA also undergoes host mitochondrial metabolism. Mouse genetic experiments identified medium-chain acyl-CoA dehydrogenase (MCAD) as an important host enzyme in the metabolism of microbiota-derived PPA and in the resulting generation of circulating metabolites, including hippurate [91]. These findings establish a host-microbial co-metabolic route in mice.

### 3.1. The PAA-PAGln-PAGly Axis in Thrombosis, Vascular Dysfunction, and Metabolic Disease

PAGln has shown reproducible associations with CVD across human cohorts. In the initial discovery and validation studies, higher circulating PAGln was associated with prevalent CVD and incident MACE, independently of established cardiovascular risk factors [90]. The human evidence is observational and therefore establishes PAGln as a risk-associated biomarker rather than a causal mediator of cardiovascular events. However, the consistency of these associations across independent populations supports further evaluation of its potential value for cardiovascular risk stratification [92,93].

Higher PAGln concentrations have also been associated with greater coronary atherosclerotic burden. In patients undergoing coronary computed tomographic angiography, circulating PAGln was independently associated with percent atheroma volume, obstructive CAD, and coronary lesion complexity, but was not associated with high-risk plaque characteristics [94]. Studies in patients with acute and chronic cardiovascular populations have further associated PAGln with multivessel disease, adverse outcomes, mortality, and hospitalization [95,96,97,98]. Human metagenomic analyses further showed that the abundance of *ppfor* and *ppdc* homologs was associated with atherosclerotic cardiovascular disease [88]; however, these human data are associative and do not establish the quantitative contribution of either pathway to PAA or PAGln production in individual patients.

Additional clinical studies have examined PAGln in relation to hypertension, arterial stiffness, heart failure, and related vascular phenotypes [99,100]. These studies extend the potential clinical relevance of PAGln across several cardiovascular phenotypes, although evidence that PAGln provides incremental predictive value beyond established clinical risk factors requires formal assessment of model discrimination, calibration, or reclassification. Similarly, elevations of PAGln in alcohol-associated dysbiosis or other cardiometabolic states demonstrate an association with altered host-microbial metabolism but do not establish that increased PAGln production directly mediates cardiovascular risk in those populations [101]. Recent experimental evidence does not support the interpretation that PAGln-associated cardiovascular risk is necessarily mediated by increased atherosclerotic plaque burden. In Western diet-fed *Apoe*^−/−^ mice, dietary PAA increased circulating phenylacetyl conjugates but reduced total atherosclerotic plaque burden in the descending and abdominal aortas of both sexes and in the brachiocephalic arteries of male mice [102]. PAA also altered lipid metabolism, decreased circulating acylcarnitines and free carnitines, and increased expression of genes involved in peroxisomal lipid metabolism. These findings suggest that the clinical association between PAGln and cardiovascular events is unlikely to be explained solely by accelerated atherosclerotic plaque accumulation. Because this experiment administered the microbial precursor PAA to mice, rather than administering PAGln to humans, the results should not be interpreted as evidence that PAGln is anti-atherosclerotic.

Evidence linking PAGln to T2D is less developed than the cardiovascular literature. Human metabolomics studies have identified PAGln among metabolites associated with T2D, diabetic complications, or metabolic dysregulation [103,104]. In obese subjects, human metabolomics studies have identified Phe-derived metabolites, including PAGln, within circulating or urinary metabolic signatures associated with adiposity [105]. These observations suggest that PAGln can reflect altered microbial and host amino acid metabolism, but they do not establish that PAGln causes obesity, insulin resistance, or microbiome remodeling.

Evidence specifically connecting PAGln to MASLD or metabolic dysfunction-associated steatohepatitis (MASH) remains limited. Multi-omics studies may implicate aromatic amino acid metabolism in MASLD [106,107], but evidence implicating the broader Phe-PAA-PAGln pathway does not necessarily demonstrate a specific role for PAGln. Consequently, it would be premature to describe the PAA-PAGln axis as “driving” metabolic liver disease or its cardiovascular complications. PAA should also be distinguished from its downstream host conjugate PAGln. In diet-induced obese mice, PAA supplementation did not alter body weight, adiposity, insulin sensitivity, hepatic steatosis, fibrosis, or markers of liver injury, but it remodeled lipid metabolism, reduced circulating triglycerides and acylcarnitines, and increased hepatic pathways associated with peroxisome proliferator-activated receptor-α (PPARα) and fatty acid oxidation [108]. These findings provide experimental evidence that PAA can alter host lipid metabolism without worsening the measured hepatic outcomes in that model; however, they should not be interpreted as establishing an equivalent effect of PAA and PAGln in humans.

**Figure 3:**
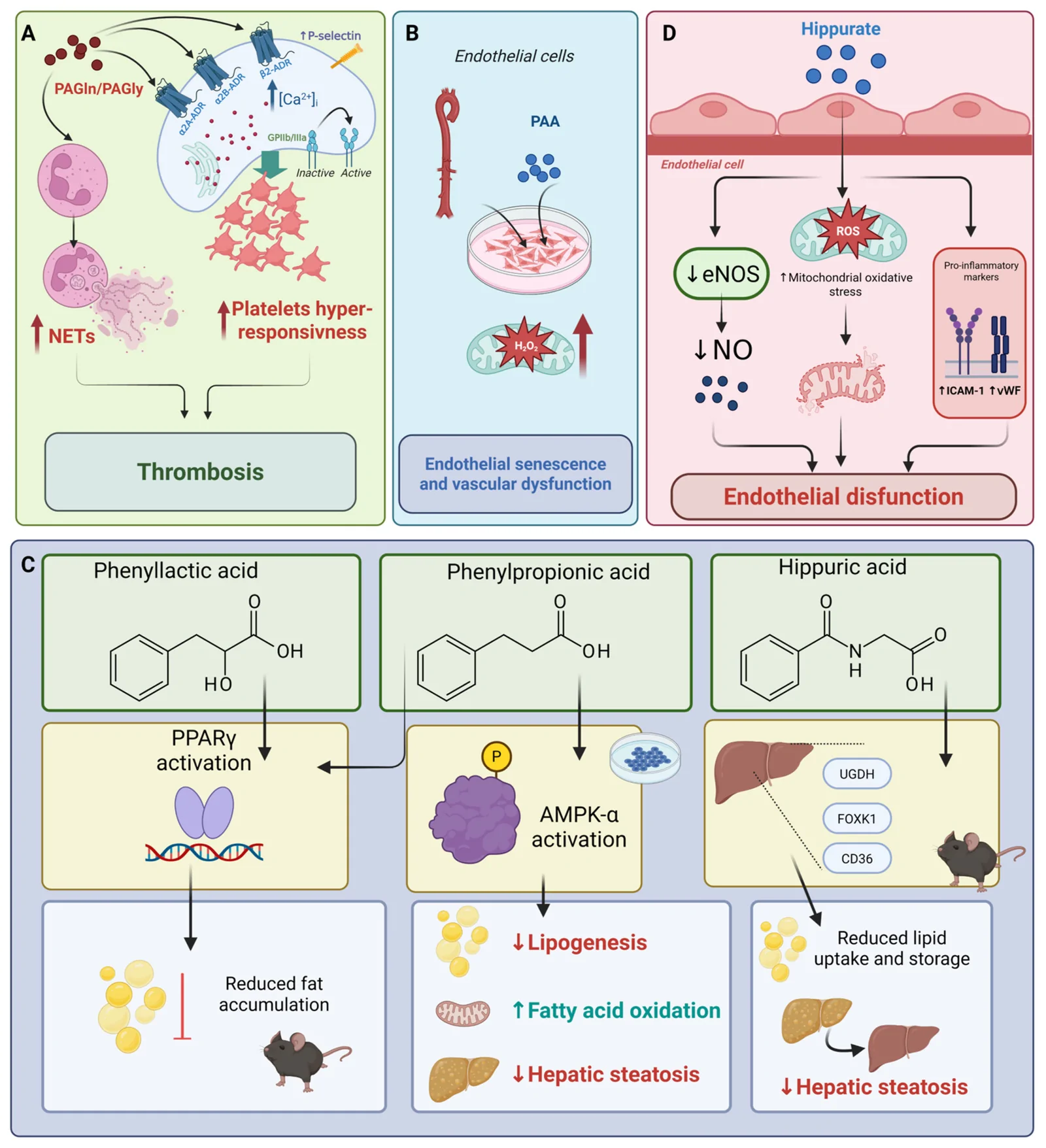
Microbial phenylalanine metabolites in cardiometabolic disease. Summary of phenylalanine-derived microbial and host-microbial co-metabolites and their experimentally supported effects on (**A**) platelet responsiveness and thrombosis, (**B**) endothelial senescence and vascular dysfunction, (**C**) metabolic and hepatic phenotypes, and (**D**) endothelial dysfunction. Evidence is derived from in vitro studies and in vivo animal models. Supporting references and details of the experimental models are provided in the corresponding sections of the main text.

Mechanistic studies provide stronger evidence that PAGln can affect thrombosis. PAGln enhances platelet responsiveness and thrombotic potential through adrenergic receptors, including α2A-, α2B-, and β2-adrenergic receptors. These findings were supported by experiments using whole blood and isolated platelets, pharmacological and genetic manipulation of adrenergic receptors, and in vivo arterial injury models. Complementary microbial genetic and gnotobiotic mouse experiments demonstrated that manipulation of microbial PAA production alters host PAGln/PAGly levels, platelet responsiveness, and thrombosis potential [90]. More recent structural and functional work identified PAGln as an endogenous negative allosteric modulator of the β2-adrenergic receptor, providing a more detailed receptor-specific mechanism for the previously observed adrenergic effects [109]. In parallel, PAGln has been linked to neutrophil extracellular trap (NET) formation in the setting of T2D and ischemic stroke. In patients with ischemic stroke, circulating PAGln correlated with NET markers and was higher in those with T2D. In a rat model of ischemic stroke, direct PAGln administration increased NET formation and was associated with greater systemic inflammation, oxidative stress, and cerebral injury [110] (Figure 3A, Table 2). Because the molecular mechanism linking PAGln to NET formation has not been established, this effect should be distinguished from the adrenergic receptor-dependent platelet pathway described above.

PAA itself may exert additional vascular effects. Experimental evidence has linked PAA exposure to endothelial-cell senescence and impaired vascular function [111] (Figure 3B, Table 2). However, these findings should be interpreted in the context of other experimental data showing that PAA does not uniformly promote adverse vascular phenotypes.

The human and experimental evidence supports a model in which circulating PAGln is associated with adverse cardiovascular outcomes across multiple cohorts, while direct experimental evidence is strongest for enhanced platelet responsiveness and thrombosis rather than accelerated atherosclerotic plaque accumulation. Its broader roles in T2D, obesity, MASLD, and MASH remain less firmly established. Thus, PAGln is best regarded as a prognostic cardiovascular biomarker and mechanistically active host-microbial co-metabolite, rather than a broadly causal cardiometabolic mediator.

### 3.2. The PPA–Hippurate Axis: Metabolic Health Marker and Context-Dependent Cardiovascular Signal

Early metabolomics studies linked altered hippurate levels to metabolic disease [112]. Cross-species metabolomic comparisons identified altered urinary hippurate among metabolic changes associated with T2D or obesity in humans and rodent models [113,114]. More recent human cohort studies have extended these observations by linking hippurate to gut microbiome diversity, diet quality, and metabolic syndrome risk. In the TwinsUK cohort, Pallister et al. [115] showed that circulating hippurate was positively associated with gut microbial diversity and dietary intake of fruits and whole grains and inversely associated with metabolic syndrome. Longitudinal analysis further demonstrated that increases in hippurate levels were associated with a reduced likelihood of metabolic syndrome. These findings highlight hippurate as a marker of the diet-microbiome-host axis, reflecting microbial diversity, dietary exposures, and metabolic health status. Longitudinal and intervention-based studies further underscore the dynamic nature of this pathway. Work by Wu et al. [116] demonstrates that microbiome-metabolome interactions, including changes in hippurate and related aromatic metabolites, are closely associated with impaired glucose control and responsiveness to lifestyle interventions. Hippurate may therefore serve as both a cross-sectional marker of metabolic state and a dynamic biomarker of metabolic responses to intervention.

Preclinical intervention studies indicate that hippurate can modify metabolic phenotypes. Brial et al. combined human multi-omics data with mechanistic mouse experiments to demonstrate that higher urinary hippurate was associated with improved metabolic health, particularly in individuals consuming diets high in saturated fat, while chronic hippurate administration improved glucose tolerance and insulin secretion in high-fat-diet-fed mice [117]. In an independent study, supplementation with the hippurate precursor PPA improved glucose tolerance in male mice. No comparable metabolic benefit was observed in female mice; instead, PPA supplementation caused increased body-weight gain and adiposity in females [108].

Recent preclinical work showed that hippurate supplementation reduced body weight and hepatic lipid accumulation in obese mice and identified a UDP-glucose 6-dehydrogenase (UGDH)–forkhead box K1 (FOXK1)–cluster of differentiation 36 (CD36) signaling mechanism contributing to reduced hepatic lipid accumulation [118]. As an upstream microbial precursor of hippurate, PPA may exert biological effects independently of its conversion to hippurate. In vitro studies indicate that PPA activates AMP-activated protein kinase α (AMPKα) and can modulate metabolic and inflammatory pathways [119]. Furthermore, in a preclinical study, phenyllactate, a direct microbial precursor of PPA, increased peroxisome proliferator-activated receptor γ (PPARγ) activation and limited diet-induced obesity [120] (Figure 3C, Table 2).

In the context of CVD, emerging evidence suggests that higher circulating hippurate can be associated with adverse cardiovascular outcomes, contrasting with the generally favorable associations reported for urinary hippurate in studies of microbial diversity and metabolic health. Human cohort studies have shown that plasma hippurate is independently associated with incident MACE even after adjustment for traditional risk factors and with advanced atherosclerosis and postoperative outcomes [43,66]. Mechanistically, hippurate may adversely affect vascular function when it accumulates systemically, particularly in the setting of CKD. Experimental studies demonstrated that hippurate reduced endothelial nitric oxide synthase (eNOS) expression and increased intercellular adhesion molecule 1 (ICAM-1) and von Willebrand factor (vWF) expression in human aortic endothelial cells through dynamin-related protein 1 (Drp1)-mediated mitochondrial fission and mitochondrial oxidative stress; endothelial dysfunction was also observed in hippurate-treated rats [121]. These findings provide experimental evidence that hippurate exposure can impair endothelial function through mitochondrial oxidative stress, although they do not establish that hippurate causes atherosclerotic plaque formation in humans. Collectively, these data indicate that the relationship between hippurate and cardiovascular risk is context-dependent. Higher circulating hippurate has been associated with adverse cardiovascular outcomes, whereas experimental accumulation of hippurate under uremic conditions can directly impair endothelial function (Figure 3D, Table 2).

In summary, the PPA–hippurate axis represents an important node in host-microbiome metabolic interplay. Hippurate is associated with greater microbial diversity, healthier dietary patterns, and favorable metabolic phenotypes in several human cohorts, while preclinical intervention studies partially support direct effects on glucose and lipid metabolism. However, these favorable metabolic associations contrast with adverse cardiovascular associations of elevated circulating hippurate and experimental endothelial toxicity under uremic conditions. Because circulating hippurate is also influenced by renal clearance and can accumulate with impaired kidney function, its concentration may reflect both microbial and host metabolism as well as host excretory capacity, underscoring the importance of renal function and disease context when interpreting these associations. PPA, an upstream microbial precursor, may additionally exert signaling effects distinct from those of hippurate.

## 4. Microbial and Host Tyr Co-Metabolism and Cardiometabolic Diseases

Tyr serves as a precursor for several biosynthetic pathways: it is converted by tyrosine hydroxylase (TH) to L-3,4-dihydroxyphenylalanine (L-DOPA), then by AADC to dopamine. Dopamine is further metabolized to norepinephrine and epinephrine via dopamine β-hydroxylase (DBH) and phenylethanolamine N-methyltransferase (PNMT), respectively [122]. Tyr is also essential for the production of the thyroid hormones triiodothyronine (T3) and thyroxine (T4) through iodination and coupling reactions within thyroglobulin, and for melanin synthesis [123,124]. In parallel, Tyr catabolism begins with transamination by tyrosine aminotransferase (TAT) to 4-hydroxyphenylpyruvic acid. This intermediate is converted by 4-hydroxyphenylpyruvate dioxygenase (HPPD) to homogentisate, which is subsequently metabolized by homogentisate 1,2-dioxygenase (HGD), maleylacetoacetate isomerase (GSTZ1), and fumarylacetoacetate hydrolase (FAH), ultimately yielding fumarate and acetoacetate [125] (Figure 1).

In the gut microbiota, 4-hydroxyphenylpyruvic acid can enter alternative pathways that generate metabolites subsequently modified by the host. As in phenylpyruvate/PAA metabolism, microbial PPDC can catalyze decarboxylation of 4-hydroxyphenylpyruvic acid to 4-hydroxyphenylacetaldehyde, which can subsequently be converted to 4-hydroxyphenylacetic acid (4-HPAA) [88]. 4-HPAA can then be metabolized to *p*-cresol by *hpdBCA*-positive bacteria. The *hpdBCA* operon encodes the HpdBCA 4-hydroxyphenylacetate decarboxylase complex, which catalyzes the terminal conversion of 4-HPAA to *p*-cresol and CO_2_. The HpdBCA complex belongs to the glycyl radical enzyme family and supports this decarboxylation under anaerobic conditions. This pathway is particularly well characterized in *Clostridioides difficile*, where *p*-cresol can be generated from Tyr-derived 4-HPAA or through uptake and metabolism of exogenous 4-HPAA [126]. Experimental studies showed that representative strains from all five *C. difficile* lineages can produce *p*-cresol through Tyr fermentation or utilization of exogenous 4-HPAA and that *p*-cresol production provides a competitive growth advantage, particularly against Gram-negative gut bacteria [126,127]. Expression of the *hpdBCA* operon is induced by 4-HPAA in a concentration-dependent manner; under the experimental conditions examined, Tyr was inefficiently converted to 4-HPAA, indicating substrate-dependent regulation of the terminal decarboxylation step rather than establishing upstream Tyr conversion as universally rate-limiting [126,127].

Following microbial production and intestinal absorption, *p*-cresol undergoes host phase II conjugation, yielding the host-microbial co-metabolites *p*-cresol sulfate (*p*CS) and *p*-cresol glucuronide (*p*CG). Sulfation is catalyzed by cytosolic sulfotransferases, including SULT1A1, using 3′-phosphoadenosine-5′-phosphosulfate (PAPS) as the sulfate donor, and represents the predominant conjugation pathway in humans. A smaller fraction of *p*-cresol undergoes glucuronidation by UGTs, generating *p*CG [128,129,130,131]. Thus, circulating *p*CS and *p*CG reflect sequential microbial generation of *p*-cresol followed by host conjugative metabolism (Figure 1, Table 1).

Alternatively, 4-hydroxyphenylpyruvic acid can enter a microbial reductive pathway leading to 4-hydroxyphenylpropionic acid (4-HPPA) through the intermediates 4-hydroxyphenyllactic acid and 4-hydroxyphenylacrylic acid [25]. Following absorption, 4-HPPA, similarly to PPA, can undergo further host metabolism, including β-oxidation and glycine conjugation, contributing to the formation of 4-hydroxyhippuric acid as a host-microbial co-metabolite [91]. A related microbial route can generate 4-vinylphenol, which can subsequently be reduced to 4-ethylphenol (4-EP). Vinylphenol reduction has been experimentally characterized in *Lactiplantibacillus plantarum* (formerly *Lactobacillus plantarum*), where the VprA reductase catalyzes the conversion of 4-vinylphenol to 4-EP. Genetic disruption of *vprA* abolished vinylphenol-reduction activity, and recombinant VprA reduced 4-vinylphenol to 4-EP, providing direct experimental evidence for this enzymatic step. Following microbial production and absorption, 4-EP undergoes host phase II metabolism, including sulfation to form the circulating host-microbial co-metabolite 4-ethylphenyl sulfate (4-EPS) [132] (Figure 1, Table 1).

### 4.1. pCS and Related Conjugates in Cardiometabolic Disease

Patients with CKD or ESKD have elevated circulating levels of *p*CS, a protein-bound host-microbial co-metabolite that accumulates as renal clearance declines and is poorly removed by conventional dialysis. Elevated *p*CS has been associated with CVD and mortality risk in multiple studies of patients with impaired renal function [133,134,135,136]. Importantly, this relationship is not restricted to advanced kidney disease. Using untargeted metabolomics as a discovery platform in individuals with primarily preserved renal function, followed by targeted quantification in an independent validation cohort, elevated circulating *p*CS was independently associated with all-cause mortality [42]. Human fecal metagenomic analyses further showed that the abundance of the microbial *hpdBCA* pathway responsible for *p*-cresol production was associated with CVD, linking circulating *p*CS not only to host renal handling but also to microbial metabolic capacity [42].

**Figure 4:**
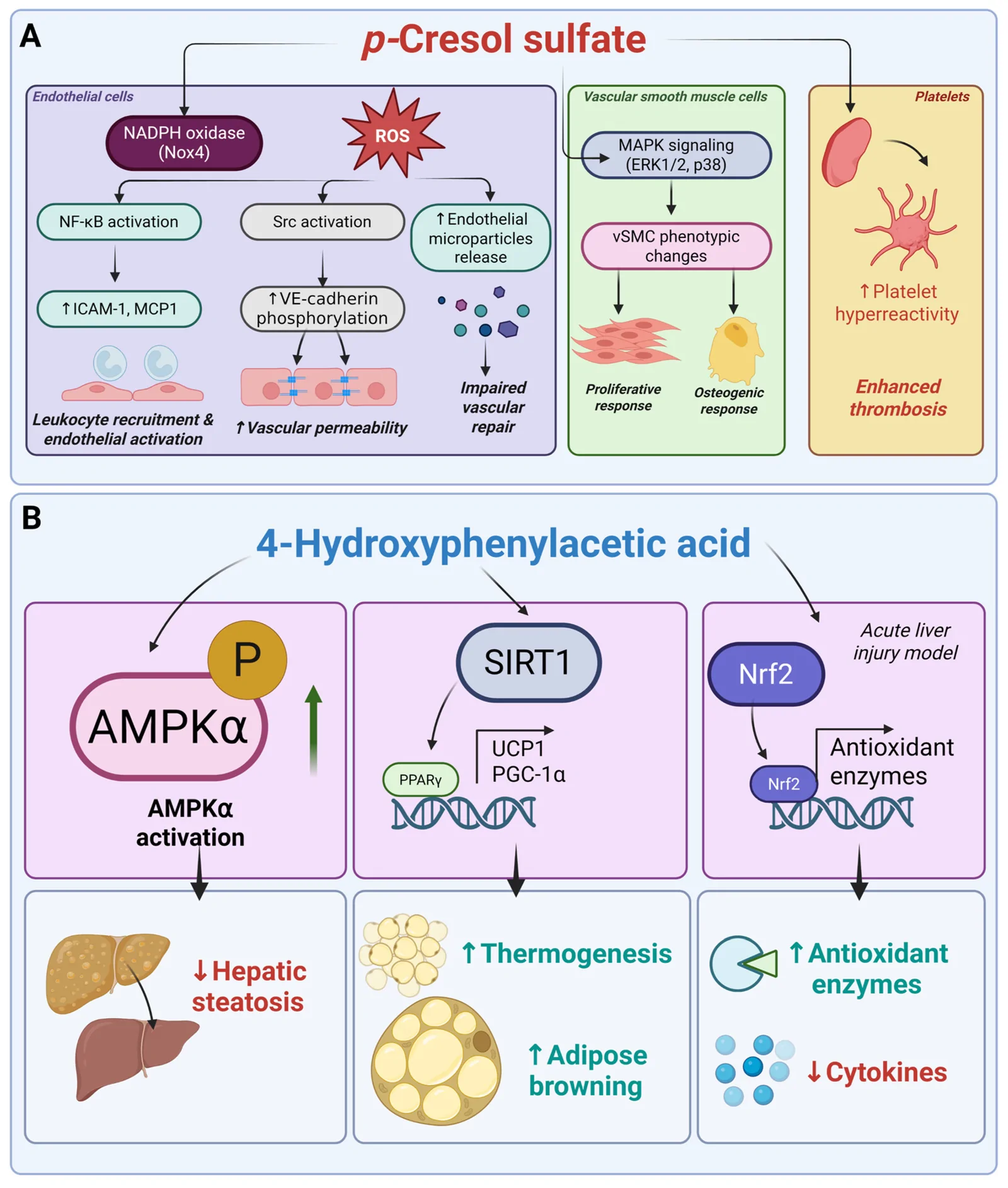
Microbial tyrosine metabolites in cardiometabolic disease. Experimentally supported effects of (**A**) the host-microbial co-metabolite *p*-cresol sulfate on vascular inflammation and dysfunction, vascular smooth muscle cell responses, and thrombosis, and (**B**) 4-hydroxyphenylacetic acid on metabolic and antioxidant pathways. The Nrf2-associated effects were demonstrated in an acute liver injury model. Evidence is derived from in vitro studies and in vivo animal models. Supporting references and details of the experimental models are provided in the corresponding sections of the main text.

Experimental studies provide evidence extending these human associations toward causality. Increasing circulating *p*CS by pharmacological administration promoted pro-thrombotic phenotypes, while microbial genetic manipulation provided complementary evidence that intestinal production of its precursor, *p*-cresol, can enhance thrombosis in vivo. Specifically, colonization of germ-free mice with genetically engineered *B. thetaiotaomicron* carrying the microbial *p*-cresol-producing pathway was sufficient to confer a pro-thrombotic phenotype, providing a direct link between microbial pathway activity and host thrombosis [42]. Beyond thrombosis, mechanistic studies in experimental systems implicate *p*CS in vascular dysfunction and remodeling. *p*CS induces oxidative stress through NADPH oxidase-dependent pathways, including Nox4, in endothelial cells and vSMCs, increasing reactive oxygen species (ROS) production and cellular injury [137,138,139]. This oxidative stress activates pro-inflammatory signaling pathways, including NF-κB, and increases expression of adhesion molecules such as ICAM-1 and chemokines such as monocyte chemoattractant protein-1 (MCP-1), promoting endothelial activation and leukocyte recruitment. In parallel, *p*CS disrupts endothelial barrier integrity through Src-dependent phosphorylation of vascular endothelial cadherin (VE-cadherin), increasing vascular permeability [140]. Experimental studies also indicate that *p*CS-induced endothelial microparticle release can impair endothelial repair [141]. In vSMCs, *p*CS has been reported to promote phenotypic changes involving MAPK signaling, including extracellular signal-regulated kinases 1 and 2 (ERK1/2) and p38, with proliferative and osteogenic responses [139] (Figure 4A, Table 2).

Evidence for metabolic effects of *p*CS is also derived predominantly from experimental studies rather than direct causal studies in humans. In patients with stable coronary artery disease, higher circulating total *p*CS was associated with a higher waist-to-hip ratio in women [55]. In cultured 3T3-L1 adipocytes, *p*CS increased intracellular reactive oxygen species production and lipid peroxidation, providing experimental evidence of oxidative stress in adipose cells [142,143]. More direct causal evidence exists for impaired glucose homeostasis: chronic administration of *p*CS to mice with normal kidney function induced insulin resistance, loss of fat mass, ectopic lipid redistribution to skeletal muscle and liver, and altered skeletal-muscle insulin signaling involving ERK1/2 activation. Complementary experiments in C2C12 myotubes demonstrated direct *p*CS-induced insulin resistance through ERK1/2 activation [143]. These findings support a direct metabolic effect of *p*CS in experimental systems, but whether *p*CS independently drives obesity, MASLD, or MASH in humans remains unresolved.

### 4.2. 4-HPAA as a Potentially Metabolically Protective Tyr-Derived Metabolite

Preclinical studies demonstrate beneficial metabolic effects of 4-HPAA in high-fat-diet-fed mouse models, including attenuation of obesity, improved glucose tolerance, and reduced hepatic steatosis [144,145].

Distinct pathways appear to contribute to these metabolic effects. Hepatic AMPKα activation was associated with the antisteatotic action of 4-HPAA [144]. In parallel, 4-HPAA modulated sirtuin 1 (SIRT1) signaling and increased markers associated with adipose tissue browning and thermogenesis, while pharmacological inhibition of SIRT1 partially attenuated its beneficial effects [145]. Additional antioxidant and cytoprotective effects have been demonstrated in an acetaminophen-induced acute liver injury model, in which 4-HPAA increased nuclear factor erythroid 2-related factor 2 (Nrf2)-associated phase II and antioxidant enzyme responses [146]. Thus, AMPKα- and SIRT1-associated effects are supported in experimental metabolic-disease models, whereas the Nrf2-associated effects derive from an acute liver-injury model and should not be interpreted as an established mechanism of the anti-obesity effects of 4-HPAA (Figure 4B, Table 2).

### 4.3. Other Microbial Metabolites Derived from Tyr in Cardiometabolic Diseases

Clinical studies have identified multiple Tyr-derived microbial metabolites, including 4-hydroxybenzoic acid, 4-hydroxyhippuric acid, and 4-hydroxyphenyllactic acid, as being associated with MACE and overall mortality; in addition, 4-EPS has been associated with increased all-cause mortality [43]. 4-Hydroxyphenyllactic acid has also been associated with cardiorenal variables in metabolically healthy individuals [147].

## 5. Microbial and Host His Co-Metabolism and Cardiometabolic Diseases

In humans, His metabolism proceeds through three major pathways. The canonical hepatic catabolic pathway converts His to glutamate through sequential reactions catalyzed by histidase (HAL), urocanase (UROC1), imidazolonepropionase (HUTI), and formiminotransferase cyclodeaminase (FTCD), thereby linking His degradation to folate-dependent one-carbon metabolism. A second pathway involves the decarboxylation of His by histidine decarboxylase (HDC) to generate histamine, an important mediator of immune, gastric, and neuronal signaling. His also contributes to the synthesis of carnosine and related histidine-containing dipeptides, which participate in intracellular pH buffering and antioxidant defense [148] (Figure 1).

In addition to these host pathways, the gut microbiota substantially expands the metabolic fate of His, generating metabolites with distinct biological activities. Among these, imidazole propionate (ImP) has received particular attention. ImP is produced through a microbial two-step pathway in which histidine ammonia-lyase (HutH) converts His to urocanate, followed by its reduction to ImP by urocanate reductase (UrdA). UrdA is a flavin-dependent, multidomain oxidoreductase containing flavin adenine dinucleotide (FAD) and flavin mononucleotide (FMN) cofactors that facilitate electron transfer during urocanate reduction. Structural studies indicate that UrdA undergoes substrate-dependent conformational changes that coordinate substrate recognition, cofactor positioning, catalysis, and product release, with active-site residues including Arg411 contributing to stabilization of catalytic intermediates [149] (Figure 1, Table 1).

Microbial His metabolism is not restricted to the ImP-producing pathway. An alternative branch begins with His decarboxylation to histamine, which can subsequently be oxidized to imidazole-4-acetaldehyde and imidazole-4-acetate through host or microbial enzyme activities. The initial reaction is catalyzed by microbial HDC, which exists in both pyruvoyl-dependent and PLP-dependent forms [150,151]. Histamine production has been identified across diverse gut-associated bacterial taxa, including *Limosilactobacillus reuteri* (formerly *Lactobacillus reuteri*) and representatives of the genera *Streptococcus*, *Clostridium*, *Bacteroides*, *Bifidobacterium*, and *Fusobacterium*, as well as members of the family Enterobacteriaceae [151]. For the pyruvoyl-dependent HDC system, *hdcA*, encoding the HDC precursor, can occur in a gene cluster with accessory genes including *hdcB*, which is required for maturation of the pyruvoyl-dependent enzyme, and *hdcP*, encoding a His/histamine antiporter [150,151].

Microbially derived histamine can subsequently undergo oxidative deamination to generate imidazole-4-acetaldehyde. In the host, this reaction is catalyzed by diamine oxidase (DAO), whereas microorganisms can employ amine oxidases capable of histamine oxidation [152]. Imidazole-4-acetaldehyde can subsequently be oxidized to imidazole-4-acetate (imidazole-4-acetic acid) by aldehyde-oxidizing enzymes. However, the specific enzymes and genetic determinants responsible for these transformations in gut microorganisms remain incompletely characterized and may vary among microbial taxa.

### ImP as a Driver of Insulin Resistance and Vascular Inflammation in Experimental Models

Initial human metabolomic studies identified higher circulating ImP concentrations in individuals with T2D and associated ImP with impaired glucose homeostasis [153]. These clinical observations were complemented by experimental studies demonstrating a direct metabolic effect of ImP. In vivo, administration of ImP to mice impaired glucose tolerance and insulin signaling, while mechanistic experiments linked these effects to activation of the p38γ/p62/mechanistic target of rapamycin complex 1 (mTORC1) pathway and inhibition of insulin receptor substrate (IRS)-dependent signaling [153].

**Figure 5:**
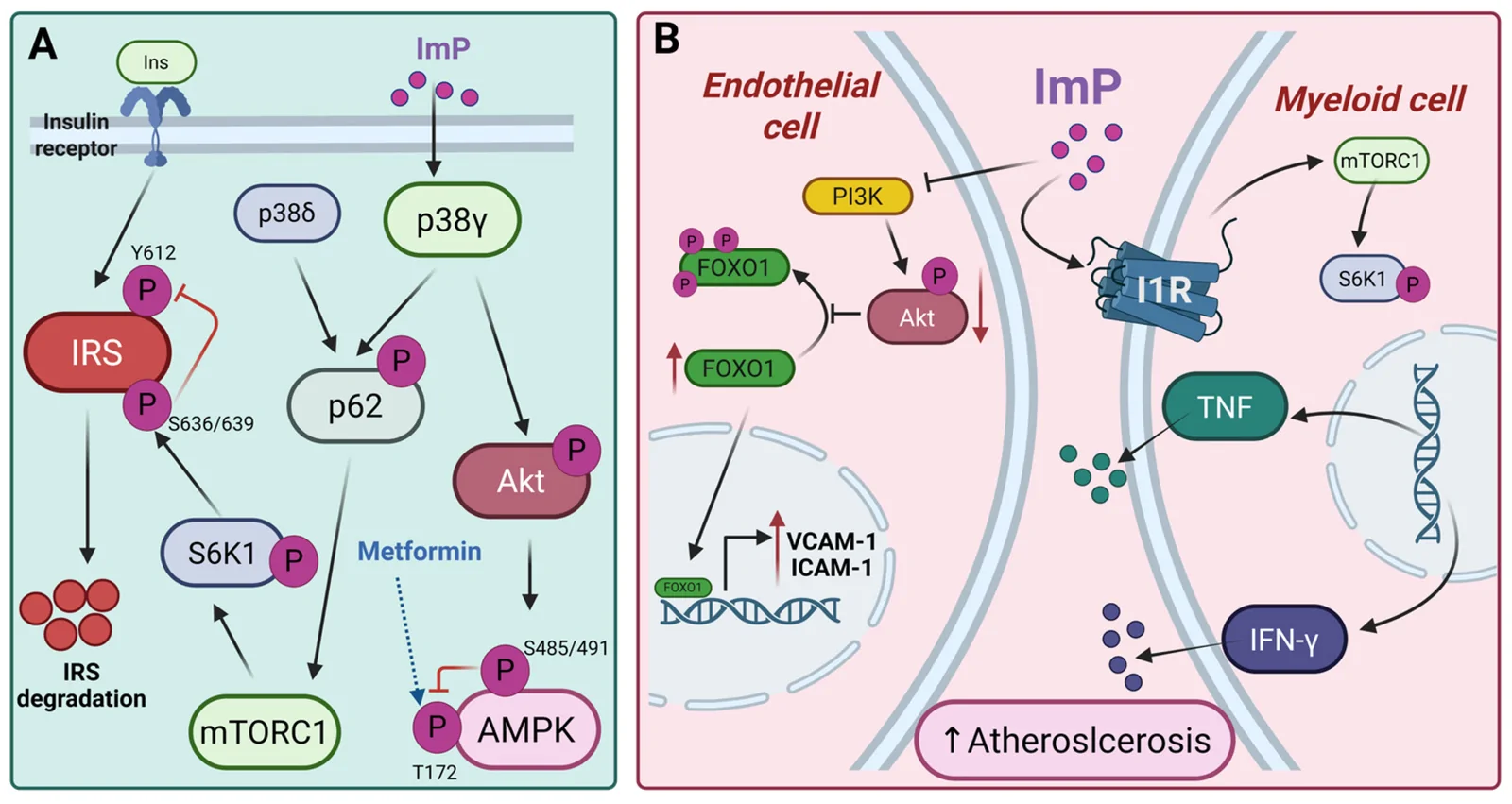
Microbial histidine metabolite imidazole propionate in cardiometabolic disease. Experimentally supported effects of imidazole propionate (ImP) on (**A**) insulin signaling and metformin response and (**B**) endothelial dysfunction, inflammation, and atherosclerosis. Evidence is derived from in vitro studies and in vivo animal models. Supporting references and details of the experimental models are provided in the corresponding sections of the main text.

A similar combination of human and experimental evidence has connected ImP with variability in the response to metformin. In humans, circulating ImP concentrations were higher in metformin-treated individuals with T2D who had elevated blood glucose, supporting an association between ImP and poorer glycemic control during metformin treatment [154]. In vivo, pretreatment of mice with ImP impaired the glucose-lowering effect of metformin. In vitro, studies in primary hepatocytes and HEK293 cells showed that ImP inhibited metformin-induced AMPK activation through a p38γ/Akt-dependent pathway involving inhibitory AMPK phosphorylation; inhibition of p38γ blocked the inhibitory effects of ImP on metformin action [154]. These findings provide experimental support for a mechanistic link between a microbiota-derived metabolite and variation in metformin responsiveness. Additional human studies have associated circulating ImP with T2D, dietary patterns, and alterations in gut microbial ecology [155] (Figure 5A, Table 2).

Beyond glucose metabolism, a growing body of human observational evidence links circulating ImP with cardiovascular and systemic metabolic phenotypes. Circulating ImP concentrations have been associated with blood pressure variability, obesity-related phenotypes, altered gut microbial diversity, and cardiometabolic risk [156,157]. Higher circulating ImP levels have also been associated with adverse cardiovascular outcomes, including heart failure and mortality [158,159], and with cardiovascular or metabolic phenotypes in coronary artery disease [160] and HIV-associated vascular disease [161]. Additional associations have been reported with albuminuria in individuals with T2D [162] and other microbiome-associated cardiometabolic comorbidities [163,164,165]. Elevated ImP has also been reported in severe COVID-19 in association with cardiac involvement [166]. Collectively, these human studies establish ImP as a biomarker associated with multiple cardiometabolic and inflammatory phenotypes but, by themselves, do not establish causality.

Experimental studies provide evidence that ImP can directly affect vascular biology. In vitro, exposure of primary human aortic endothelial cells to ImP reduced endothelial cell migration and formation of tube-like structures and was accompanied by altered angiogenesis-related transcriptional programs and reduced phosphoinositide 3-kinase (PI3K)/Akt signaling. In vivo, ImP treatment impaired vascular re-endothelialization following carotid artery injury in mice, supporting a direct detrimental effect on endothelial repair [167]. More recent work has provided substantially stronger evidence for a causal role of ImP in atherosclerosis [168]. This study combined human observational data with experimental models. In humans, circulating ImP was associated with the extent of atherosclerosis in two independent cohorts, an observation that remains associative. In vivo, administration of ImP to atherosclerosis-prone mice was sufficient to promote atherosclerosis without altering the lipid profile and was accompanied by systemic and local immune activation and inflammation. Mechanistic experiments identified the imidazoline-1 receptor (I1R), also known as nischarin, in myeloid cells as a mediator of these effects. Blocking the ImP–I1R axis inhibited atherosclerosis induced by either ImP or a high-cholesterol diet in mice [168] (Figure 5B, Table 2). Whereas the human data establish an association between ImP and atherosclerotic burden, the in vivo animal experiments provide evidence that ImP can actively drive atherogenesis and identify I1R as a mechanistic mediator.

Experimental studies further indicate that the biological actions of ImP extend beyond classical metabolic and vascular pathways. In diabetic wound healing, in vivo experiments in mice showed that ImP impaired wound repair and angiogenesis. Complementary mechanistic experiments demonstrated that ImP inhibited spinster homolog 2 (SPNS2)-mediated sphingosine-1-phosphate (S1P) secretion and Rho signaling, thereby impairing angiogenic responses in human umbilical vein endothelial cells in vitro [169]. ImP has also been reported to promote diabetic kidney disease through Toll-like receptor 4 (TLR4)-dependent inflammatory signaling [170]. In contrast to the adverse effects reported in other experimental settings, ImP improved adipocyte lipid metabolism and attenuated high-fat diet–induced obesity through PPAR-dependent signaling in a separate preclinical study [171].

Taken together, the available evidence supports a distinction between circulating ImP as a human disease-associated biomarker and ImP as an experimentally defined bioactive metabolite. Human cohort studies consistently associate ImP with metabolic and cardiovascular disease phenotypes, whereas cell-based and animal studies provide mechanistic evidence that ImP can directly perturb insulin signaling, metformin action, endothelial repair, inflammatory responses, atherogenesis, and tissue-specific metabolic pathways.

## 6. Therapeutic Modulation of Microbial Aromatic Amino Acid Metabolism

Microbial aromatic amino acid metabolism has emerged as a potential therapeutic target because many metabolites discussed throughout this Review have been associated with inflammation, vascular pathology, metabolic dysfunction, and renal disease. However, translating these observations into effective therapies remains challenging. Metabolite concentrations are determined by interacting factors, including dietary substrate availability, microbial community composition, microbial enzymatic capacity, host metabolic processing, tissue distribution, and renal clearance. Consequently, altering the concentration of one compound does not necessarily produce clinical benefit or accurately reflect changes in the broader metabolic network.

An additional challenge is distinguishing biomarkers from causal mediators. Some metabolites, including PAGln and ImP, are supported by mechanistic studies linking microbial pathways to specific host receptors and signaling pathways. For many others, evidence remains largely associative. Thus, an intervention-induced change in a metabolite concentration should be interpreted as evidence of target engagement rather than proof of therapeutic efficacy unless it is accompanied by meaningful physiological effects or improved clinical outcomes. Future interventions must therefore be evaluated not only for their ability to alter metabolite levels but also for their impact on disease progression, cardiovascular outcomes, metabolic health, and long-term safety.

### 6.1. Translational Challenges and Safety Considerations

Despite growing enthusiasm for microbiome-targeted therapies, several important biological and clinical challenges deserve consideration. First, aromatic amino acid metabolism operates as an interconnected network rather than as a set of isolated pathways. Suppressing one route may redirect substrates toward alternative microbial or host reactions, generating metabolites with distinct or even opposing biological effects. Because Trp, Phe, Tyr, and His each feed multiple competing routes, an intervention designed to reduce one metabolite may unintentionally increase others.

Second, many metabolites exhibit context-dependent effects. Associations observed in CKD, obesity, T2D, MASLD, or cardiovascular disease may not generalize across diseases, tissues, or physiological states. Metabolites considered potentially detrimental in one context may participate in physiological signaling in another. Accordingly, long-term modification of these pathways could produce consequences not apparent in short-duration experiments.

Third, most studies measure changes in metabolite concentrations rather than clinical outcomes. Demonstrating that an intervention lowers IS, *p*CS, PAGln, or ImP is evidence of target engagement but does not establish improved clinical outcomes. This distinction is particularly important because few microbiome-targeted interventions have undergone large-scale clinical outcome testing.

### 6.2. Diet as a Regulator of Microbial Aromatic Amino Acid Metabolism

**Figure 6:**
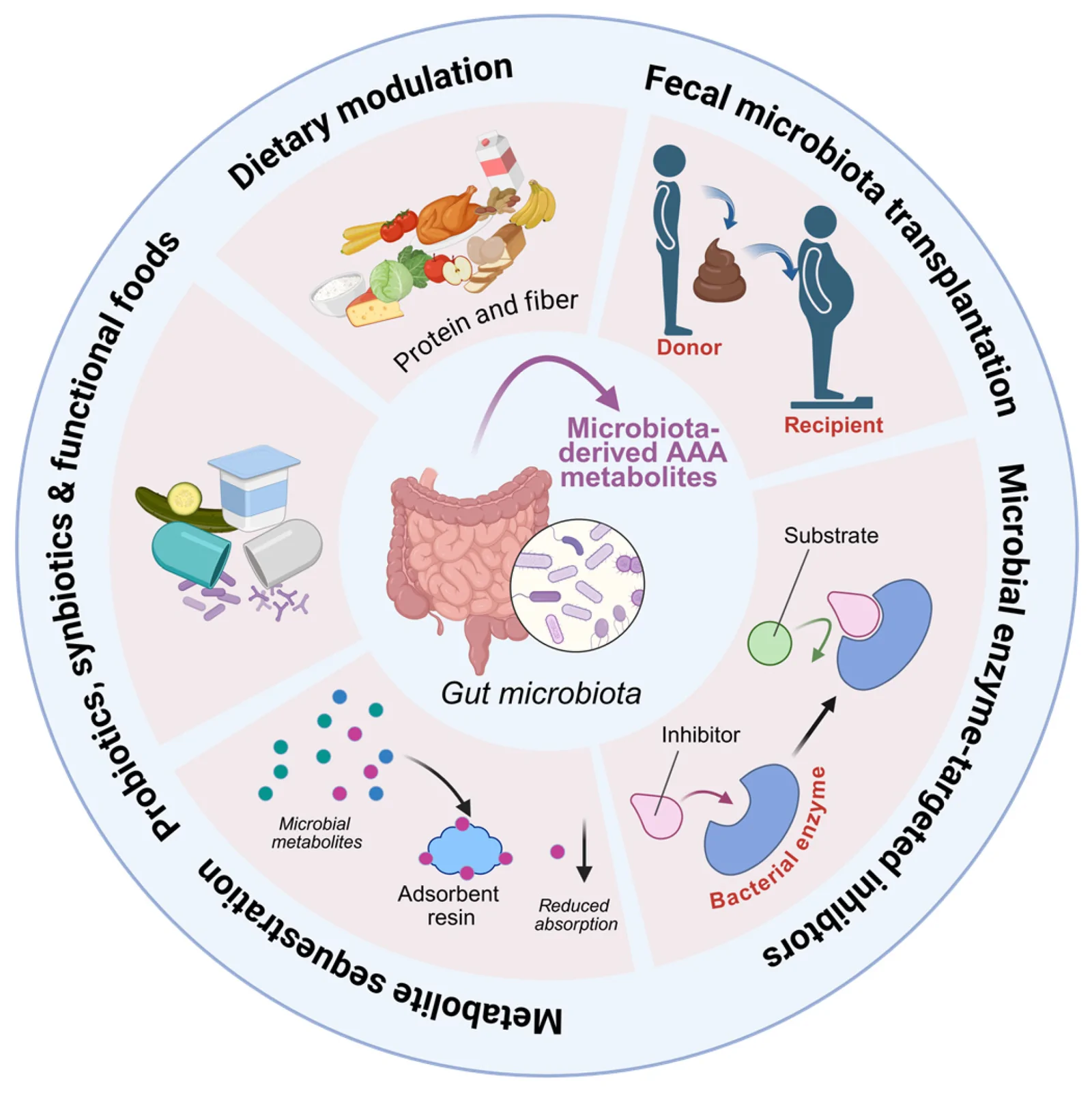
Therapeutic strategies for modulating gut microbiota-derived aromatic amino acid metabolism. Dietary interventions, probiotics, synbiotics and functional foods, fecal microbiota transplantation (FMT), microbial enzyme-targeted inhibitors, and metabolite sequestration represent complementary approaches to modifying microbial metabolism or reducing exposure to downstream metabolites. These strategies differ in pathway specificity, consistency of effects on circulating metabolites, and level of supporting evidence. Human intervention evidence is available for some dietary, probiotic, FMT, and related microbiota-directed approaches, whereas selective microbial enzyme-targeted strategies remain predominantly preclinical. Changes in circulating metabolite concentrations indicate biological or target engagement but do not by themselves establish clinical benefit.

Diet represents the most clinically accessible strategy for modifying microbial aromatic amino acid metabolism (Figure 6). Dietary proteins are major substrates for microbial amino acid metabolism and directly influence the pool of precursor amino acids reaching the colon. Beyond substrate supply, diet also shapes the taxonomic and functional composition of the gut microbiota. Clinical intervention studies support an effect of dietary protein intake on circulating microbial metabolites. In a randomized crossover study involving 32 patients with CKD not receiving dialysis, a very-low-protein diet (0.3 g/kg body weight/day) supplemented with ketoanalogues reduced circulating IS by 37% compared with a low-protein diet (0.6 g/kg/day) [172]. Similarly, in individuals with T2D, a 12-week high-protein diet increased fasting PAGln, IS, and IAA levels, whereas IPA levels decreased compared with an isocaloric low-protein diet [173], indicating that a high-protein diet can shift the circulating metabolite profile toward higher concentrations of potentially harmful metabolites.

However, diet alone may not determine metabolite production. In human studies, circulating ImP levels were more closely associated with microbiome features, such as reduced gene richness and enrichment of ImP-producing pathways, than with dietary His intake [155], suggesting that the metabolic capacity of the microbiome influences the conversion of His into ImP. Human and mouse supplementation studies further show that His availability can increase ImP production in a microbiota-dependent manner [174], indicating that ImP production reflects an interaction between substrate availability and microbial metabolic capacity rather than dietary intake alone.

Protein fermentation is also highly dependent on the surrounding luminal environment. Increased availability of carbohydrates favors saccharolytic metabolism, leading to greater SCFA production, lower colonic pH, and suppression of proteolysis [7]. Important mechanistic evidence comes from experimental rather than clinical intervention studies. Using defined microbial communities in vitro, human fecal cultures, and gnotobiotic mice, Sinha et al. demonstrated that dietary fiber can redirect microbial Trp metabolism through metabolic interactions among bacterial species [175]. Fiber-degrading *B. thetaiotaomicron* released monosaccharides that induced catabolite repression in indole-producing *Escherichia coli*, thereby suppressing indole production and increasing Trp availability to *C. sporogenes* for production of ILA and IPA. Moreover, the fiber-dependent reduction in indole production was reproduced in human fecal cultures and fecal-microbiota-transplanted gnotobiotic mice. These experiments indicate that the fate of Trp can be governed by substrate-dependent microbial competition and metabolic regulation rather than by the abundance of Trp-metabolizing organisms alone [175]. Consistent with the direction of these experimental findings, human dietary studies have associated fiber-rich diets with increased circulating IPA in older adults with preserved renal function [176].

Clinical trials of fiber supplementation, however, indicate that effects differ among microbiota-derived uremic toxins. Fermentable fiber interventions can reduce *p*CS, whereas effects on IS have been less consistent. For example, a randomized clinical trial of resistant starch reported a reduction in *p*CS without a significant reduction in IS [177]. More complex dietary interventions that simultaneously increase fiber and reduce protein intake, including Mediterranean-style diets, have produced moderate reductions in both IS and *p*CS together with changes in gut microbiome composition [178].

Although human dietary studies therefore provide evidence that protein and fiber intake can alter circulating microbiota-related metabolites, broad protein restriction may not be appropriate for all populations. Older adults, individuals with frailty or sarcopenia, and patients with increased protein requirements may experience adverse effects from prolonged restriction. Likewise, high-fiber interventions can influence gastrointestinal physiology, nutrient absorption, medication bioavailability, and production of non-target microbial metabolites. Therefore, dietary manipulation should be viewed as a systems-level intervention rather than a selective method for targeting a single pathway.

Importantly, repeated studies demonstrate substantial heterogeneity in metabolic responses. Identical dietary interventions may produce very different metabolite profiles because of differences in microbiome composition, enzyme abundance, medications, host genetics, renal function, and baseline eating patterns. These observations highlight the need for personalized dietary approaches informed by microbial function rather than generalized dietary recommendations alone.

### 6.3. Fecal Microbiota Transplantation (FMT) and Metabolite Remodeling

FMT represents a broad strategy for altering microbial community structure and function. Its effects on aromatic amino acid-derived metabolites have been examined in both preclinical models and human intervention studies, although the endpoints and strength of evidence differ substantially (Figure 6). Preclinical studies provide direct evidence that manipulating the gut microbiota can alter aromatic amino acid-derived metabolites. In mouse models, antibiotic-mediated depletion of the gut microbiota reduces microbial metabolites including IS and *p*CS, supporting their microbial origin, whereas microbial recolonization can restore metabolite production. In an adenine-induced CKD mouse model, transplantation of microbiota from healthy donor mice increased microbial diversity and reduced plasma *p*CS, but did not improve kidney function [179,180].

Human evidence in CKD remains limited. In a single-center, double-blind, randomized, placebo-controlled trial, 28 patients with diabetes and/or hypertension and CKD stages 2–4 received oral FMT or placebo capsules for six months [181]. Fewer patients in the FMT group showed CKD progression than in the placebo group, and serum creatinine and urea nitrogen remained stable in the FMT group. FMT was also associated with changes in gut microbial composition, including decreased abundance of Bacillota (formerly Firmicutes) and Actinomycetota (formerly Actinobacteria) and increased abundance of Bacteroidota (formerly Bacteroidetes), Pseudomonadota (formerly Proteobacteria), and *Roseburia* spp. Importantly, however, this clinical trial did not establish that the observed renal effects were mediated by reductions in IS, *p*CS, or other aromatic amino acid-derived metabolites. This small, single-center intervention study therefore provides preliminary human evidence that oral FMT can modify gut microbiome composition and may influence renal measures, but it does not establish pathway-specific therapeutic efficacy [181].

Human FMT studies in metabolic disease further demonstrate that successful modification of microbial composition does not necessarily translate into predictable changes in microbial metabolite production. In individuals with MASLD, repeated allogeneic vegan-donor FMT has been reported to alter gut microbial profiles, PAGln levels, and hepatic DNA methylation compared with autologous FMT [182]. These observations indicate that FMT can be accompanied by changes in microbial composition, circulating microbial metabolites, and host hepatic molecular profiles. However, the direction and magnitude of these effects appear to be pathway- and context-dependent, underscoring the difficulty of predicting specific metabolite responses following ecosystem-level microbiome manipulation.

FMT is therefore fundamentally different from interventions designed to inhibit a specific microbial enzyme or metabolic pathway. It introduces a complex microbial ecosystem containing numerous metabolic functions, making its downstream effects inherently difficult to predict. Potential concerns include transmission of undesirable microorganisms or functions, variable donor-microbiota engraftment, ecological instability, and unintended changes in non-target microbial pathways. Moreover, the long-term consequences of reshaping aromatic amino acid metabolism through ecosystem-level microbiome manipulation have not been established in controlled human studies. Consequently, although preclinical studies demonstrate that microbiome manipulation can alter aromatic amino acid-derived metabolite production, current human evidence does not establish consistent or selective lowering of these metabolites or pathway-mediated clinical benefit following FMT.

### 6.4. Probiotic and Functional Food Strategies to Modulate Aromatic Amino Acid Metabolism

Probiotics, prebiotics, synbiotics, and related functional-food strategies have been evaluated in human intervention studies primarily for their effects on circulating microbial metabolites, microbiome composition, intestinal-barrier measures, and inflammatory markers; direct evidence of pathway-specific clinical efficacy remains limited (Figure 6). The observed effects, however, are heterogeneous and depend on the type of intervention. In a human intervention study in patients with CKD, supplementation with *Lactobacillus acidophilus* KBL409 reduced serum IS and was accompanied by changes in intestinal permeability and inflammatory markers [183]. However, these clinical observations do not establish that *L. acidophilus* KBL409 directly reduces IS production, because changes in circulating IS may result from broader alterations in microbial community function, intestinal barrier properties, or host handling of the metabolite. Moreover, the effect is not consistently observed across clinical trials. A systematic review and meta-analysis of 14 randomized controlled trials involving 513 participants with CKD found no significant overall reduction in circulating IS with microbiota-directed therapies, whereas circulating *p*CS was significantly reduced [184]. Subgroup analyses indicated that the reduction in *p*CS was observed with prebiotic and synbiotic interventions but not with probiotics alone.

Human intervention data also suggest that probiotics can alter circulating Trp-derived metabolites, although the underlying mechanism cannot be inferred from these clinical associations alone. In a randomized, double-blind, placebo-controlled multicenter trial of 63 healthy adults ≥65 years of age, participants received *Bifidobacterium bifidum* BGN4 and *Bifidobacterium longum* BORI or placebo for 12 weeks. Probiotic supplementation altered circulating metabolites involved in Trp metabolism, and microbiota-derived IPA increased 1.91-fold in the probiotic group and was associated with changes in gut bacterial profiles [185]. Importantly, these findings demonstrate an effect of probiotic supplementation on circulating IPA in humans but do not establish that the administered *Bifidobacterium* strains directly produced IPA. The clinical component should therefore be interpreted as evidence of an intervention-associated change in microbial Trp metabolism rather than proof of a specific microbial mechanism.

Functional foods represent a related but conceptually distinct approach. Fermented foods can deliver viable microorganisms together with a complex food matrix, whereas prebiotic foods or ingredients provide substrates that can alter microbial metabolism without introducing new organisms. Accordingly, changes in aromatic amino acid-derived metabolites following consumption of probiotic or fermented functional foods may arise from interactions among the administered organisms, dietary substrates, and the resident microbiota rather than direct production of the metabolite by the supplemented strain. This distinction is particularly relevant to IPA, because its production depends on microbial metabolic capacity as well as substrate availability and community context. Thus, evidence obtained with defined probiotic capsules should not automatically be generalized to fermented foods, and conversely, metabolic effects of functional foods cannot necessarily be attributed to their constituent probiotic organisms.

Probiotics and synbiotics should therefore be viewed primarily as ecosystem-level rather than pathway-selective interventions. Their metabolic effects depend on strain persistence, interactions with resident organisms, cross-feeding, and substrate availability.

More pathway-selective approaches under preclinical investigation include rationally selected microbial consortia, combinations of organisms with defined dietary substrates, and engineered strains designed to direct aromatic amino acid metabolism toward or away from specific metabolites. These approaches provide mechanistic proof of concept but have not yet established efficacy or safety in controlled human studies.

### 6.5. Enzyme-Targeted Inhibitors and Metabolite Sequestration

Selective inhibition of microbial metabolic enzymes is being investigated as a preclinical strategy for reducing production of specific gut microbial metabolites while sparing homologous host pathways. Current studies have primarily assessed enzyme inhibition, microbial metabolite production, circulating metabolite concentrations, and downstream phenotypes in biochemical systems, microbial cultures, and animal models. Mechanism-based strategies, including transition-state analogs and substrate-activated irreversible inhibitors, have shown high specificity and potency in preclinical experimental systems. For clinical translation, candidate compounds would ideally act as non-lethal metabolic modulators, exhibit minimal systemic absorption, remain localized within the intestinal lumen, and target conserved microbial enzymatic features while minimizing effects on microbial viability or community structure. Together, these characteristics define a precision microbiome-targeting paradigm that differs fundamentally from conventional antimicrobial therapies, emphasizing functional modulation rather than microbial eradication.

Small-molecule approaches to reduce gut microbial production of indole and *p*-cresol have increasingly focused on the inhibition of selected bacterial enzymes. As indole is generated exclusively through bacterial tryptophanase activity, recent work has identified mechanism-based inhibitors such as the transition-state analog (3S)-ALG 05, which acts as a broad-spectrum inhibitor of microbial tryptophanases and effectively reduces intestinal indole production and circulating IS levels in animal models while preserving microbial viability [186]. Similarly, HpdBCA-dependent *p*-cresol production can be inhibited by small molecules such as 4-hydroxyphenylacetonitrile, which achieves near-complete suppression (~99%) of *p*-cresol generation in vitro [127]. Nitazoxanide (NTZ), an antiparasitic drug that inhibits microbial pyruvate:ferredoxin oxidoreductase, reduced PAA production through the PFOR pathway in vitro but did not inhibit production through the PPDC pathway. In mice, however, NTZ did not reduce plasma or urinary PAA, PAGln, or PAGly levels, although it lowered *p*CS [88]. Given that *p*-cresol and PAA share common upstream metabolic steps (Figure 1), and that NTZ can inhibit both enzymatic activity and the growth of certain bacteria, the reduction in *p*CS may reflect alterations in microbial community composition rather than direct inhibition of the *p*-cresol pathway.

Despite their conceptual appeal, enzyme-targeted strategies face several important challenges. Aromatic amino acid metabolism is characterized by substantial pathway redundancy, with multiple organisms capable of producing similar metabolites through distinct enzymatic routes. As demonstrated for PAA production, inhibition of one pathway may leave alternative routes unaffected. Long-term safety considerations also remain largely unexplored. Chronic suppression of microbial metabolic activities may alter nutrient utilization, ecological competition, microbial adaptation, and host physiology in ways that cannot be predicted from short-term studies. For these reasons, most enzyme-targeted approaches should currently be viewed as promising preclinical proof-of-concept strategies rather than clinically validated therapies.

In parallel, the oral adsorbent AB-2004 has been shown to reduce circulating levels of gut-derived phenolic metabolites, including metabolites from the 4-EP/4-EPS pathway, as well as *p*CS and IS, in preclinical models and in a Phase 1b/2a clinical study in adolescents with autism, demonstrating target engagement; improvements in behavioral symptoms were also reported [187]. However, its efficacy has not been tested in individuals with CKD or in the context of cardiometabolic diseases. Another adsorbent, AST-120, reduced fecal *p-*cresol and indole levels in preclinical studies [188]. AST-120 has also been evaluated in several randomized clinical trials in CKD, including a multicenter study in advanced disease [189] and the Evaluating Prevention of Progression in Chronic Kidney Disease (EPPIC) trial program [190]. However, these trials did not demonstrate consistent improvements in renal outcomes, highlighting the distinction between preclinical evidence of metabolite modulation and demonstrated clinical efficacy (Figure 6).

### 6.6. Clinical Trial Gaps and Future Priorities

Despite rapidly expanding mechanistic knowledge, translation of aromatic amino acid metabolism research into clinical medicine remains at an early stage. Most available evidence comes from observational studies, animal models, mechanistic investigations, or short-duration intervention trials. Relatively few studies have directly tested whether targeted modification of aromatic amino acid pathways improves clinically meaningful outcomes.

Several important gaps remain. First, many studies rely on surrogate metabolic endpoints without assessing disease progression or patient benefit. Second, investigations often fail to simultaneously assess microbial composition, microbial enzymatic capacity, metabolite production, and host responses, thereby limiting causal interpretation. Third, marked interindividual variability arising from diet, medications, genetics, disease state, renal function, and microbiome composition complicates translation.

Future clinical trials should integrate pathway-specific biomarkers, longitudinal metabolomics, microbial functional profiling, isotope tracing, and predefined clinical endpoints. Such studies will be essential for determining whether modulation of aromatic amino acid metabolism produces benefits beyond biomarker changes and ultimately improves cardiometabolic health.

## 7. Conclusions and Future Directions

Microbial metabolism of aromatic amino acids represents an important interface linking diet, gut microbial ecology, host metabolism, and cardiometabolic disease. Across Trp-, Phe-, Tyr-, and His-derived pathways, a consistent theme emerges: metabolite biology is fundamentally context-dependent. The physiological consequences of a given metabolite are shaped not only by its concentration, but also by its microbial source, pathway of production, host transformation, tissue distribution, duration of exposure, renal clearance, inflammatory state, and disease context. As a result, individual metabolites should not be classified simply as beneficial or detrimental. Rather, they exist within dynamic metabolic networks whose outputs depend on both microbial and host factors.

An important challenge moving forward is distinguishing biomarkers from mediators. Many aromatic amino acid-derived metabolites are strongly associated with obesity, T2D, MASLD, CVD, heart failure, and CKD. However, detection of an association does not establish causality, just as demonstration of a biological effect following metabolite administration does not necessarily define the physiological role of endogenously produced metabolite pools. Future studies should therefore prioritize approaches that integrate microbial genetics, isotope tracing, functional enzymology, receptor perturbation, and longitudinal human investigations to determine when altered pathway activity is sufficient to drive disease and when metabolites primarily reflect underlying physiological states.

Equally important is a shift from purely taxonomic descriptions of the microbiome toward pathway-level and flux-based analyses. The abundance of a microbial species does not necessarily predict metabolite production because pathway expression depends on dietary substrate availability, microbial competition, cross-feeding interactions, and environmental conditions within the gut ecosystem. Advances in metagenomics, metatranscriptomics, metaproteomics, metabolomics, and stable-isotope approaches will allow more precise characterization of the microbial enzymes and pathways responsible for aromatic amino acid metabolism in vivo. Such studies will be essential for understanding why individuals with similar microbiota compositions often exhibit markedly different metabolic phenotypes.

Although this Review focuses primarily on intestinal microbial metabolism, microbiome-cardiovascular interactions are not restricted to the gut. Emerging evidence supports an oral–gut–heart axis in which oral dysbiosis may influence cardiovascular disease through microbial translocation, immune and inflammatory signaling, and metabolic cross-talk. Periodontal dysbiosis involving taxa such as *Porphyromonas gingivalis*, *Fusobacterium nucleatum*, *Aggregatibacter actinomycetemcomitans*, and *Tannerella forsythia* has been associated with inflammatory and cardiovascular phenotypes; however, direct involvement of their aromatic amino acid metabolism in cardiovascular disease remains uncertain [191,192,193,194,195]. Future studies integrating oral and gut microbial functional profiling with metabolomics and pathway-specific analyses will be important for determining whether aromatic amino acid metabolism represents a shared mechanistic link across these microbial niches.

These considerations also have important implications for therapeutic translation. Dietary modification, prebiotics, probiotics, synbiotics, FMT, enzyme-targeted inhibitors, and metabolite sequestration strategies each provide opportunities to modulate aromatic amino acid metabolism. However, these approaches differ substantially in specificity, reproducibility, and clinical maturity. Changes in circulating metabolite levels should therefore not be interpreted as evidence of therapeutic benefit without demonstration of pathway engagement and meaningful improvements in physiological or clinical outcomes. Future intervention studies should incorporate comprehensive assessments of microbial function, metabolite production, host responses, and disease-relevant endpoints while accounting for dietary patterns, renal function, and baseline microbiome composition.

Ultimately, a precision-medicine framework for microbiome-targeted therapy will require identifying the microbial pathways most relevant to and active in a given individual, understanding the environmental and host factors that regulate those pathways, and selectively modifying harmful metabolic activities while preserving beneficial microbial functions. By integrating diet, microbial enzymatic capacity, metabolite flux, and host physiology, future research may enable personalized strategies that move beyond broad microbiome manipulation toward targeted control of aromatic amino acid metabolism in cardiometabolic disease.

## Figures and Tables

**Table 1:** Representative microbial pathways involved in aromatic amino acid metabolism.

Amino Acid	Microbial Product	Microbial Enzyme(s)	Representative Experimentally Supported Taxa	Downstream Host-Microbial Co-Metabolite(s)
Trp	Indole	TnaA	*Escherichia coli*; other TnaA-positive gut bacteria	IS; IG
Trp	Tryptamine	TDC	*C. sporogenes*; *M. gnavus*; *B. hansenii*; *E. asparagiformis*; *F. nexilis*	N/A
Trp	IPA	FldH, FldA, FldBC, FldI, and AcdA	*C. sporogenes*	N/A
Trp	IAA	OFORs	*I. bartlettii*; *B. hydrogenotrophica*	N/A
		TMO; IaaH	*L. reuteri*; Canonical TMO/IaaH pathway characterized principally in plant-associated bacteria	
Phe	PAA	PPFOR; PPDC-dependent pathway	*B. thetaiotaomicron* (PPFOR); *P. mirabilis* (PPDC-dependent pathway)	PAGln, predominantly in humans; PAGly, predominantly in rodents
Phe	PPA	FldH, FldA, FldBC, FldI, and AcdA	*C. sporogenes*	Hippurate
Tyr	4-HPAA	PPDC-dependent pathway	Taxonomic contributions remain incompletely defined	N/A
Tyr	*p*-Cresol	HpdBCA	*C. difficile*	*p*CS; *p*CG
Tyr	4-HPPA	FldH, FldA, FldBC, FldI, and AcdA	*C. sporogenes*	4-Hydroxyhippurate
Tyr	4-EP	VprA	*L. plantarum*	4-EPS
His	ImP	HutH; UrdA	UrdA-positive gut bacterial taxa	N/A
His	Histamine	HDC	*L. reuteri*	ImAA; formation may involve host or microbial oxidation of histamine.

Microbial products are generated directly through the microbial pathways shown, whereas downstream host-microbial co-metabolites generally result from subsequent host metabolism of absorbed microbial products; ImAA formation may involve host or microbial oxidation. Abbreviations: AcdA, acyl-CoA dehydrogenase; FldA, acyl-CoA transferase; FldBC, aryl-lactoyl-CoA dehydratase complex; FldH, phenyllactate/aryl-lactate dehydrogenase; FldI, activating enzyme of the aryl-lactoyl-CoA dehydratase complex; HDC, histidine decarboxylase; His, histidine; HpdBCA, 4-hydroxyphenylacetate decarboxylase complex; HutH, histidine ammonia-lyase; IAA, indole-3-acetic acid; IaaH, indole-3-acetamide hydrolase; IG, indoxyl glucuronide; ImAA, imidazole-4-acetic acid; ImP, imidazole propionate; IPA, indole-3-propionic acid; IS, indoxyl sulfate; N/A, not applicable; OFOR, 2-oxoacid:ferredoxin oxidoreductase; PAA, phenylacetic acid; PAGln, phenylacetylglutamine; PAGly, phenylacetylglycine; *p*CG, *p*-cresol glucuronide; *p*CS, *p*-cresol sulfate; Phe, phenylalanine; PPA, phenylpropionic acid; PPFOR, phenylpyruvate:ferredoxin oxidoreductase; PPDC, phenylpyruvate decarboxylase; TDC, tryptophan decarboxylase; TMO, tryptophan 2-monooxygenase; TnaA, tryptophanase; Trp, tryptophan; Tyr, tyrosine; UrdA, urocanate reductase; VprA, vinylphenol reductase A; 4-EP, 4-ethylphenol; 4-EPS, 4-ethylphenyl sulfate; 4-HPAA, 4-hydroxyphenylacetic acid; 4-HPPA, 4-hydroxyphenylpropionic acid. Supporting references for the human associations, mechanistic studies, and experimental models summarized here are provided in the corresponding sections of the main text.

**Table 2:** Human cardiometabolic associations and experimentally supported mechanisms of selected aromatic amino acid-derived microbial metabolites and host-microbial co-metabolites. Arrows indicate the direction of human observational associations and do not imply causality. Experimental designations identify the model supporting each mechanism. Microbial genetic studies are shown separately to distinguish manipulation of microbial metabolite production from direct metabolite administration.

Metabolite	Human Observational Association(s)	Experimentally Supported Mechanism(s) and Model	Microbial Genetic Validation of Metabolite Production and Host Phenotype
Indole	Limited evidence	AhR-associated epithelial signaling; increased tight-junction expression and improved intestinal barrier integrity; reduced NF-κB-associated inflammatory signaling [V]	NE
IS	↑ Cardiovascular events and mortality in CKD/ESKD; ↑ adverse vascular outcomes	ROS–p38 MAPK-dependent platelet activation and thrombosis [V,A]; tissue-factor stabilization [V]; endothelial oxidative stress and reduced NO bioavailability [V]; AhR-dependent angiogenic dysfunction [V,PP]; OATP2B1–DLL4/Notch-mediated macrophage inflammation and atherosclerosis [V,A,PP]	Engineered *B. thetaiotaomicron* expressing tryptophanase altered host IS level and affected a prothrombotic phenotype [MG]
IPA	↓ Incident T2D and adverse metabolic phenotypes; ↓ low-grade inflammation; ↓ CAD/CVD mortality; ↓ circulating concentrations in CAD and HFpEF	Increased macrophage cholesterol efflux through miR-142-5p/ABCA1 signaling and reduced atherosclerosis [V,A]; AhR–SIRT3/NAD^+^ signaling in HFpEF [A]; regulation of glucose and metabolic homeostasis [A]	*C. sporogenes fldC*/*acdA* pathway mutants established microbial control of circulating IPA [MG]
IAA	↑ Cardiovascular events and mortality in CKD; ↓ obesity- and T2D-associated phenotypes	Endothelial oxidative stress and AhR/p38 MAPK/NF-κB inflammatory signaling [V]; reduced hepatic steatosis and inflammation in metabolic liver-disease models [A]; AhR-dependent regulation of intestinal mucin sulfation [V,A]	*L. reuteri iaaM* deletion reduced IAA production and abolished the intestinal phenotype produced by the WT strain in mice [MG]
PAGln/PAGly	↑ Prevalent CVD and coronary atherosclerotic burden; ↑ incident MACE; ↑ HF, mortality, and adverse cardiovascular outcomes	α2A-, α2B-, and β2-adrenergic receptor-dependent enhancement of platelet responsiveness and thrombosis [V,A,PP]; negative allosteric modulation of β2-adrenergic receptor signaling [V]; increased NET formation and cerebral injury in experimental stroke [A]	*C. sporogenes porA*/*fldH* mutants altered host PAGln/PAGly level and affected a prothrombotic phenotype [MG]
PAA	↑ Hepatic steatosis in nondiabetic obese women	Endothelial senescence and vascular dysfunction [V]; lipid metabolic remodeling and reduced plaque burden in experimental atherosclerosis [A]; altered hepatic lipid metabolism [A]	NE
Hippurate	↓ Metabolic syndrome and adverse metabolic phenotypes in studies of urinary hippurate; ↑ MACE and advanced atherosclerosis	Improved glucose tolerance and insulin secretion [A]; UGDH–FOXK1–CD36-associated reduction in hepatic lipid accumulation [A]; Drp1-dependent mitochondrial oxidative stress and endothelial dysfunction under elevated systemic exposure [V,A]	NE
PPA	Limited evidence	AMPK-associated metabolic signaling [V]; modulation of obesity and glucose homeostasis with sex-dependent responses [A]	NE
*p*CS	↑ Cardiovascular outcomes and mortality in CKD/ESKD; ↑ adverse metabolic and vascular phenotypes	Nox4-associated oxidative stress and endothelial/vSMC injury [V]; Src–VE-cadherin-mediated endothelial permeability [V]; ERK1/2-dependent insulin resistance [V,A]; increased thrombosis [A]	Engineered *B. thetaiotaomicron* expressing *hpdBCA* increased host *p*CS level and affected a prothrombotic phenotype [MG]
4-HPAA	Limited evidence	AMPKα-associated reduction in hepatic steatosis [A]; SIRT1-associated adipose browning, thermogenesis, and metabolic improvement [A,PP]; Nrf2-mediated antioxidant signaling in experimental acute liver injury [A]	NE
4-EPS	↑ All-cause mortality; specific cardiometabolic evidence remains limited	Cardiometabolic mechanism remains incompletely defined	NE
ImP	↑ T2D and impaired glucose homeostasis; ↑ association with poorer glycemic control during metformin treatment; ↑ CAD; ↑ HF, mortality, and adverse cardiovascular outcomes	p38γ/p62/mTORC1-mediated impairment of insulin signaling [A]; p38γ/Akt-dependent inhibition of metformin-induced AMPK activation [V,A,PP]; impaired endothelial migration, angiogenesis, and vascular repair [V,A]; myeloid I1R-dependent inflammation and atherogenesis [A,PP]; SPNS2/S1P–Rho-dependent impairment of angiogenesis and diabetic wound healing [V,A]	NE

All human evidence summarized in this table is observational and does not establish causality or mechanism. Human observational association designations: ↑, higher metabolite concentrations associated with greater disease prevalence, severity, or risk; ↓, higher metabolite concentrations associated with lower disease prevalence, severity, or risk. Experimental evidence designations: [V], in vitro or ex vivo evidence; [A], in vivo animal-model evidence; [MG], microbial genetic manipulation; [PP], genetic or pharmacological perturbation of the host pathway/mechanism. Preclinical mechanistic findings should not be interpreted as established clinical effects in humans. Abbreviations: ABCA1, ATP-binding cassette transporter A1; AhR, aryl hydrocarbon receptor; AMPK, AMP-activated protein kinase; CAD, coronary artery disease; CKD, chronic kidney disease; CVD, cardiovascular disease; DLL4, delta-like canonical Notch ligand 4; Drp1, dynamin-related protein 1; ERK1/2, extracellular signal-regulated kinases 1 and 2; ESKD, end-stage kidney disease; HF, heart failure; HFpEF, heart failure with preserved ejection fraction; IAA, indole-3-acetic acid; I1R, imidazoline-1 receptor; ImP, imidazole propionate; IPA, indole-3-propionic acid; IS, indoxyl sulfate; MACE, major adverse cardiac events; MAPK, mitogen-activated protein kinase; NE, not established; NET, neutrophil extracellular trap; NF-κB, nuclear factor kappa B; NO, nitric oxide; Nox4, NADPH oxidase 4; OATP2B1, organic anion-transporting polypeptide 2B1; PAA, phenylacetic acid; PAGln, phenylacetylglutamine; PAGly, phenylacetylglycine; PPA, phenylpropionic acid; *p*CS, *p*-cresol sulfate; ROS, reactive oxygen species; S1P, sphingosine-1-phosphate; SIRT1, sirtuin 1; SIRT3, sirtuin 3; SPNS2, spinster homolog 2; T2D, type 2 diabetes; UGDH, UDP-glucose 6-dehydrogenase; VE-cadherin, vascular endothelial cadherin; vSMC, vascular smooth muscle cell; 4-EPS, 4-ethylphenyl sulfate; 4-HPAA, 4-hydroxyphenylacetic acid. Supporting references for the human associations, mechanistic studies, and experimental models summarized here are provided in the corresponding sections of the main text.

## Data Availability

No new data were created or analyzed in this study. Data sharing is not applicable to this article.

## Abbreviations

The following abbreviations are used in this manuscript:

3HAO	3-hydroxyanthranilate 3,4-dioxygenase
4-EP	4-ethylphenol
4-EPS	4-ethylphenyl sulfate
4-HPAA	4-hydroxyphenylacetic acid
4-HPPA	4-hydroxyphenylpropionic acid
5-HT	5-hydroxytryptamine
5-HTP	5-hydroxytryptophan
5-HIAA	5-hydroxyindole-3-acetic acid
AADC	aromatic L-amino acid decarboxylase
ABCA1	ATP-binding cassette transporter A1
AcdA	acyl-CoA dehydrogenase
AhR	aryl hydrocarbon receptor
ALDH	aldehyde dehydrogenase
AMPK	AMP-activated protein kinase
ArAT	aromatic amino acid aminotransferase
ASCVD	atherosclerotic cardiovascular disease
BH_4_	tetrahydrobiopterin
CAD	coronary artery disease
CD36	cluster of differentiation 36
CKD	chronic kidney disease
CRP	C-reactive protein
CVD	cardiovascular disease
CYP1B1	cytochrome P450 family 1 subfamily B member 1
CYP2E1	cytochrome P450 2E1
DAO	diamine oxidase
DBH	dopamine β-hydroxylase
DHPR	dihydropteridine reductase
DLL4	delta-like canonical Notch ligand 4
Drp1	dynamin-related protein 1
eNOS	endothelial nitric oxide synthase
EPIC	European Prospective Investigation into Cancer and Nutrition
EPPIC	Evaluating Prevention of Progression in Chronic Kidney Disease
ERK1/2	extracellular signal-regulated kinases 1 and 2
ESKD	end-stage kidney disease
FAD	flavin adenine dinucleotide
FAH	fumarylacetoacetate hydrolase
FldA	acyl-CoA transferase
FldBC	aryl-lactoyl-CoA dehydratase complex
FldH	phenyllactate/aryl-lactate dehydrogenase
FldI	activating enzyme of the phenyllactate dehydratase complex
FMN	flavin mononucleotide
FMT	fecal microbiota transplantation
FOXK1	forkhead box K1
FTCD	formiminotransferase cyclodeaminase
GLNAT	glutamine N-acyltransferase
GLYAT	glycine N-acyltransferase
GSTZ1	glutathione S-transferase zeta 1
HAL	histidase
HDC	histidine decarboxylase
HEMO	Hemodialysis study
HF	heart failure
HFpEF	heart failure with preserved ejection fraction
HGD	homogentisate 1,2-dioxygenase
His	histidine
HPPD	4-hydroxyphenylpyruvate dioxygenase
HpdBCA	4-hydroxyphenylacetate decarboxylase complex
hsCRP	high-sensitivity C-reactive protein
HutH	histidine ammonia-lyase
HUTI	imidazolonepropionase
HUVEC	human umbilical vein endothelial cell
I1R	imidazoline-1 receptor
IAA	indole-3-acetic acid
IaaH	indole-3-acetamide hydrolase
IAld	indole-3-aldehyde
IAM	indole-3-acetamide
ICAM-1	intercellular adhesion molecule 1
IDO1	indoleamine 2,3-dioxygenase 1
IG	indoxyl glucuronide
ILA	indole-3-lactic acid
ImAA	imidazole-4-acetic acid
ImP	imidazole propionate
IPA	indole-3-propionic acid
IS	indoxyl sulfate
KMO	kynurenine 3-monooxygenase
L-DOPA	L-3,4-dihydroxyphenylalanine
MACE	major adverse cardiac events
MAOs	monoamine oxidases
MAPK	mitogen-activated protein kinase
MASLD	metabolic dysfunction-associated steatotic liver disease
MASH	metabolic dysfunction-associated steatohepatitis
MCAD	medium-chain acyl-CoA dehydrogenase
MCP-1	monocyte chemoattractant protein 1
miR-142-5p	microRNA-142-5p
mTORC1	mechanistic target of rapamycin complex 1
NAD^+^	nicotinamide adenine dinucleotide
NADPH	nicotinamide adenine dinucleotide phosphate
NET	neutrophil extracellular trap
NF-κB	nuclear factor kappa B
NO	nitric oxide
Nox4	NADPH oxidase 4
Nrf2	nuclear factor erythroid 2–related factor 2
NTZ	nitazoxanide
OATP2B1	organic anion transporting polypeptide 2B1
OFOR	2-oxoacid:ferredoxin oxidoreductase
PAA	phenylacetic acid
PAGln	phenylacetylglutamine
PAGly	phenylacetylglycine
PAH	phenylalanine hydroxylase
PAPS	3′-phosphoadenosine-5′-phosphosulfate
pCG	p-cresol glucuronide
pCS	p-cresol sulfate
PFOR	pyruvate:ferredoxin oxidoreductase
Phe	phenylalanine
PI3K	phosphoinositide 3-kinase
PLP	pyridoxal 5′-phosphate
PNMT	phenylethanolamine N-methyltransferase
PPA	phenylpropionic acid
PPAR	peroxisome proliferator-activated receptor
PPDC	phenylpyruvate decarboxylase
PPFOR	phenylpyruvate:ferredoxin oxidoreductase
QPRT	quinolinate phosphoribosyltransferase
ROS	reactive oxygen species
S1P	sphingosine-1-phosphate
SCFA	short-chain fatty acid
SIRT1/3	sirtuin 1/3
SPNS2	spinster homolog 2
SULT1A1	sulfotransferase 1A1
T2D	type 2 diabetes
T3	triiodothyronine
T4	thyroxine
TAT	tyrosine aminotransferase
TDC	tryptophan decarboxylase
TDO	tryptophan 2,3-dioxygenase
TH	tyrosine hydroxylase
TLR4	Toll-like receptor 4
TMO	tryptophan 2-monooxygenase
TnaA	tryptophanase
TPH1	tryptophan hydroxylase 1
TPH2	tryptophan hydroxylase 2
Trp	tryptophan
Tyr	tyrosine
UGDH	UDP-glucose 6-dehydrogenase
UGT	UDP-glucuronosyltransferase
UROC1	urocanase
UrdA	urocanate reductase
VE-cadherin	vascular endothelial cadherin
VprA	vinylphenol reductase A
vSMC	vascular smooth muscle cell
vWF	von Willebrand factor
